# Spatial and temporal heterogeneity in the lineage progression of fine oligodendrocyte subtypes

**DOI:** 10.1186/s12915-022-01325-z

**Published:** 2022-05-25

**Authors:** Markus M. Hilscher, Christoffer Mattsson Langseth, Petra Kukanja, Chika Yokota, Mats Nilsson, Gonçalo Castelo-Branco

**Affiliations:** 1 Science for Life Laboratory, Department of Biochemistry and Biophysics, Stockholm University, 171 65 Solna, Sweden; 2 Laboratory of Molecular Neurobiology, Department Medical Biochemistry and Biophysics, Karolinska Institutet, Biomedicum, 17177 Stockholm, Sweden

**Keywords:** Oligodendrocytes, Lineage progression, Spatial transcriptomics, In situ sequencing, Cortex, Corpus callosum, Spinal cord

## Abstract

**Background:**

Oligodendrocytes are glial cells that support and insulate axons in the central nervous system through the production of myelin. Oligodendrocytes arise throughout embryonic and early postnatal development from oligodendrocyte precursor cells (OPCs), and recent work demonstrated that they are a transcriptional heterogeneous cell population, but the regional and functional implications of this heterogeneity are less clear. Here, we apply in situ sequencing (ISS) to simultaneously probe the expression of 124 marker genes of distinct oligodendrocyte populations, providing comprehensive maps of the corpus callosum, cingulate, motor, and somatosensory cortex in the brain, as well as gray matter (GM) and white matter (WM) regions in the spinal cord, at postnatal (P10), juvenile (P20), and young adult (P60) stages. We systematically compare the abundances of these populations and investigate the neighboring preference of distinct oligodendrocyte populations.

**Results:**

We observed that oligodendrocyte lineage progression is more advanced in the juvenile spinal cord compared to the brain, corroborating with previous studies. We found myelination still ongoing in the adult corpus callosum while it was more advanced in the cortex. Interestingly, we also observed a lateral-to-medial gradient of oligodendrocyte lineage progression in the juvenile cortex, which could be linked to arealization, as well as a deep-to-superficial gradient with mature oligodendrocytes preferentially accumulating in the deeper layers of the cortex. The ISS experiments also exposed differences in abundances and population dynamics over time between GM and WM regions in the brain and spinal cord, indicating regional differences within GM and WM, and we found that neighboring preferences of some oligodendroglia populations are altered from the juvenile to the adult CNS.

**Conclusions:**

Overall, our ISS experiments reveal spatial heterogeneity of oligodendrocyte lineage progression in the brain and spinal cord and uncover differences in the timing of oligodendrocyte differentiation and myelination, which could be relevant to further investigate functional heterogeneity of oligodendroglia, especially in the context of injury or disease.

**Supplementary Information:**

The online version contains supplementary material available at 10.1186/s12915-022-01325-z.

## Background

Classically, oligodendrocytes (OLs) have been considered a homogeneous population in the central nervous system (CNS). Recent advances in single-cell RNA sequencing (scRNA-seq) have allowed systematic studies of the transcriptome in individual cells, which has changed the paradigm of cellular classification [1]. At least twelve distinct OL populations can be distinguished in the CNS [2]. If these transcriptionally distinct populations are indeed different cell types or rather states remains a recurring question in the field [3], and their spatial distribution could give further insights into the heterogeneity of OLs.

The OL lineage presents itself as a continuum of oligodendrocyte precursor cells (OPCs), committed OPCs (COPs), newly formed OLs (NFOLs), myelin-forming OLs (MFOLs), and mature OLs (MOLs) [2]. In our previous study, focusing on a limited number of markers by applying RNASCOPE in situ hybridization, we have shown that OPC, MOL2, and MOL5/6 take spatial preference in the CNS, and the abundance changes with age [4]. More precisely in the neocortex and corpus callosum, the OPC population decreases with age while the MOL5/6 population increases in both regions [4]. Also, in the spinal cord, the MOL5/6 population increases with age, and it shows a spatial preference for gray matter, while the MOL2 population shows a spatial preference for white matter [4].

Most recently, novel spatial transcriptomics methods with high multiplexy have been developed that can in principle map the location of each gene and each cell [5, 6]. Multiple ex situ and in situ spatial transcriptomics technologies are now available. In situ sequencing (ISS) is a targeted, image-based approach that allows the precise mapping of genes in their natural context and offers cellular resolution [7]. Here, we applied probabilistic cell typing by in situ sequencing (pciSeq) [8] to target OLs for the first time, leveraging scRNA-seq data to assign detected transcripts to segmented cells and, subsequently, cells to cell types. We used the full advantages of ISS by probing 124 marker genes that characterize all twelve previously described populations of the OL lineage and the vascular and leptomeningeal cells (VLMCs) [2]. We investigated multiple brain and spinal cord tissue sections and found region- and age-related differences in the oligodendrocyte lineage progression, uncovering the differences in the timing of oligodendrocyte differentiation and myelination in these areas.

## Results

### In situ sequencing allows the detection of major OL populations

**Fig. 1:**
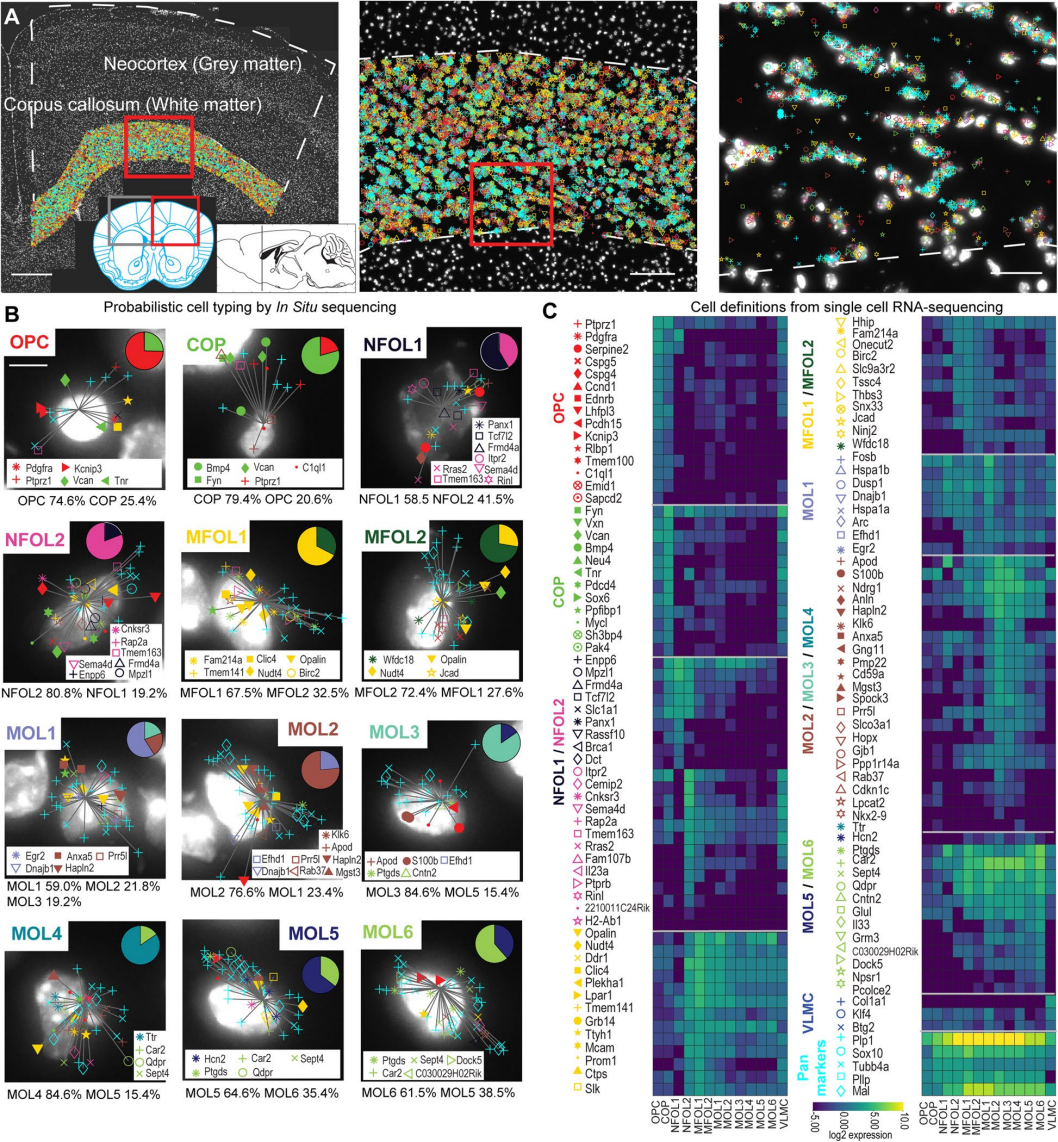
Oligodendrocyte types in the neocortex (CTX), grey matter (GM), corpus callosum (CC), and white matter (WM). **a** Maps of 124 genes in coronal mouse brain sections (P60) targeted by in situ sequencing, here highlighting the CC. Three different zoom levels are shown with white dashed lines denoting the outlines for CTX and CC. The red boxes mark the positions of the zoom-ins. The scale bars are 500 μm (left), 100 μm (middle), and 15 μm (right). **b** Individual example cells of P60 CC and their gene assignments to cells are shown. The cells are classified based on single-cell RNA sequencing (scRNA-seq) data and represented as pie charts where the angle of each slice is proportional to the likelihood of the cell being of a certain type and the size of the pie chart is indicative of the number of transcripts. Examples shown are median cells for each cell population, i.e., cells with the median count of the number of transcripts being assigned within each type. The most differentially expressed genes of the example cells are shown in the insets, and the probability for each putative cell type from pciSeq (probabilistic cell typing by in situ sequencing) is listed below the example cells. The scale bar is 5 μm. **c** The 124 genes are grouped based on the expression values in the scRNA-seq data. The color of the assigned gene symbols represents the cell population with the highest expression of the respective gene. The individual genes in the heatmaps are ranked by their expression values

We performed in situ sequencing (ISS) in postnatal (P10), juvenile (P20), and young adult (P60) mouse brain sections as well as thoracic spinal cord sections, and we quantified the OL lineage populations in their white matter (WM) vs. gray matter (GM), respectively. In the first batch, we mapped 6 P20- and 6 P60-hemisected coronal brains including the neocortex (CTX) and corpus callosum (CC) from 8 mice in total (4 biological replicates each). In the second batch, we mapped 4 P10-hemisected coronal sections from an additional 4 mice. We also mapped 4 P20 and 4 P60 spinal cord sections. The gene maps of CC (WM) in a P60 brain section are shown in Fig. 1A, with the colored symbols representing unique transcripts. The 124 genes were selected from single-cell RNA sequencing data from [2] based on the median expression values to target major markers for the oligodendrocyte populations (Fig. 1C). The color of the assigned gene symbols represents the cell population with the highest expression of the respective gene; however, we used a probabilistic gene to cell type assignment strategy. Applying pciSeq [8], we assigned detected transcripts to segmented cells and, subsequently, based on their genetic signature cells to cell populations. Thereby, each cell is represented as a pie chart, with the angle of each slice being proportional to the cell type probability. The highest probability in the pie chart defines the final cell population. The mean probability, i.e., the mean certainty, for the OL assignments was 77%, with similar probabilities for the different cell populations (Additional file 1: Fig. S1A). Only cells that express *Plp1* as a PAN marker for OLs were considered for downstream analysis. Example cells that are cells with the median count of genes per population are shown in Fig. 1B. On average, 13.4 ± 4.1 transcripts and 9.4 ± 0.4 distinctive genes per cell were measured in the OL populations in P10 brains, 14.6 ± 2.1 transcripts and 10.0 ± 0.1 distinctive genes per cell were measured in P20 brains, and 17.4 ± 2.7 transcripts and 9.8 ± 0.1 distinctive genes per cell were measured in P60 brains (Additional file 1: Fig. S1B, C). Heatmaps of the log2 expression per gene and the most differentially expressed genes per cell population are shown in Additional file 2: Fig. S2, emphasizing that many OL-associated genes are shared between OL populations but that the composition of the genes per OL population is unique, allowing us to map these populations with pciSeq.

**Fig. 2:**
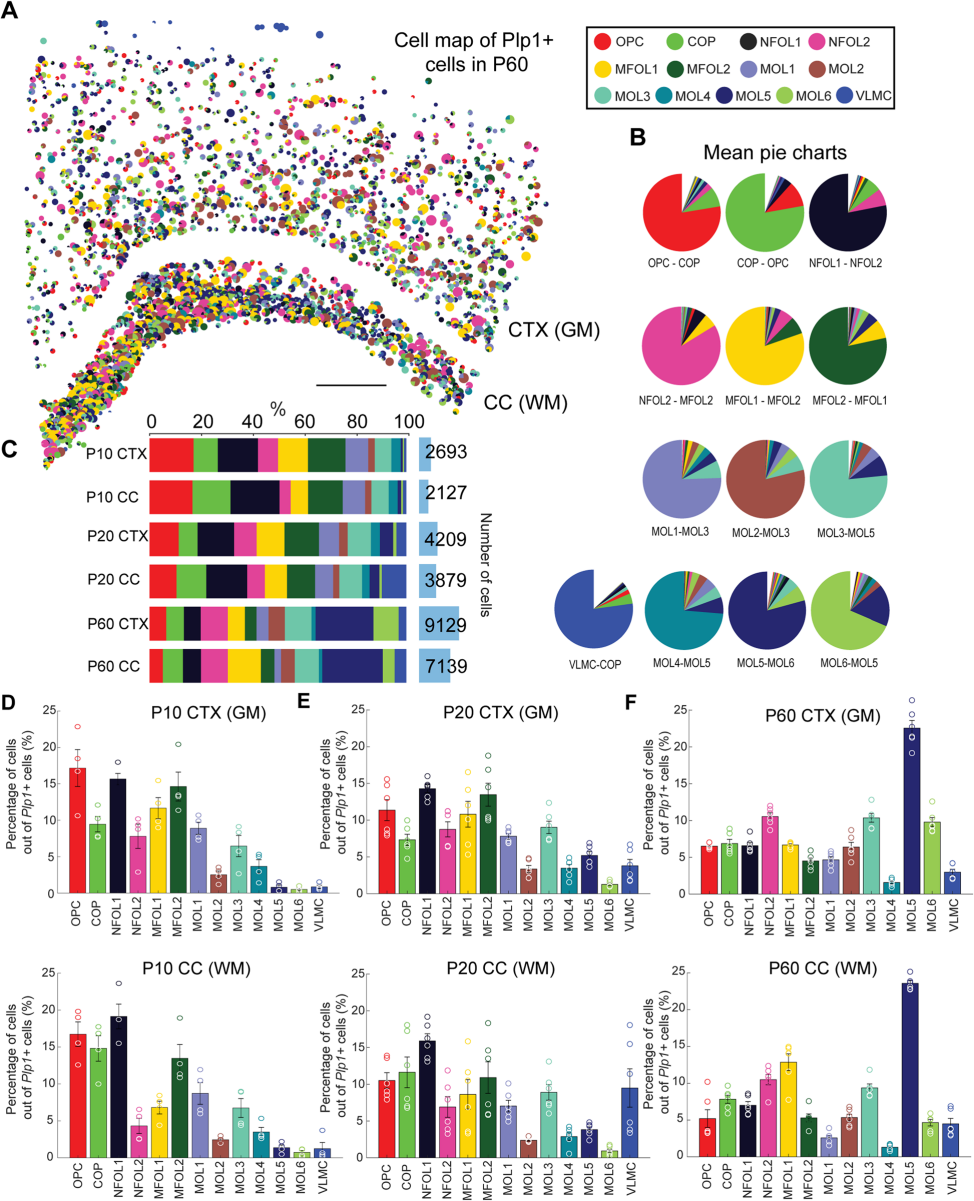
Oligodendrocyte map and abundances in P10, P20, and P60 CTX (GM) and CC (WM). **a** Spatial map of *Plp1*+ cell types in a P60 coronal mouse brain section including neocortex (CTX) and corpus callosum (CC). The scale bar is 500 μm. **b** The mean pie charts for P60 (CTX and CC joined) generated by counting how often a certain OL population was called with other OL populations. *Note*: Considering the top pies within the pie charts (caption below), the charts highlight the oligodendrocyte lineage from OPC to MOL6. **c** The number of captured oligodendrocytes as well as the relative distribution of the oligodendrocyte types per age condition and brain region (*n* = 4 P10 tissue sections, *n* = 6 P20 tissue sections, *n* = 6 P60 tissue sections). **d** The mean relative number of OL types for P10 CTX (top) and CC (bottom). The counts are normalized as counts per class divided by the total counts of *Plp1*+ cells per tissue for CTX and CC. Circles represent the counts in individual sections, and error bars represent the standard error of the mean (*n* = 4 hemisected tissue sections from 4 mice). **e** Same as (**d)** for P20 (*n* = 6 hemisected tissue sections from 4 mice). **f** Same as (**d)** for P60 (*n* = 6 hemisected tissue sections from 4 mice)

Representative cell maps of OL populations are shown in Fig. 2A (P60) and Additional file 3: Fig. S3. The size of each pie chart in Fig. 2A is indicative of the number of assigned transcripts. We also generated the mean pie charts counting how often a certain OL population was called with other OL populations (Fig. 2B). The mean pie charts per OL population over the brain tissues (*n* = 6 tissue sections) reflect the progression of OL lineage very well, with OPCs often being shared with COPs, NFOLs with MFOLs, and MFOLs with MOLs. The relatedness of the six MOL populations can also be seen in the mean confusion matrix of the cell populations during the probabilistic assignment (Additional file 4: Fig. S4).

### Myelination advances earlier in GM (CTX) than in WM (CC) in the brain

In total, we captured 2693 OLs in P10 CTX, 2127 OLs in P10 CC, 4209 OLs in P20 CTX, 3879 OLs in P20 CC, 9129 OLs in P60 CTX, and 7139 OLs in P60 CC (*n* = 4 P10 tissue sections, *n* = 6 P20 tissue sections, *n* = 6 P60 tissue sections, Fig. 2C). For P10, we captured a tissue area of 3.2 ± 0.3 mm^2^ of CTX and a tissue area of 0.8 ± 0.1 mm^2^ of CC, yielding an OL density of 207.2 ± 16.3 cells/mm^2^ in CTX and an increased OL density of 648.4 ± 134.6 cells/mm^2^ in CC (*p* = 0.002, *n* = 4 tissue sections). For P20, we captured a tissue area of 3.6 ± 0.3 mm^2^ of CTX and a tissue area of 0.9 ± 0.1 mm^2^ of CC, yielding an OL density of 193.5 ± 31.8 cells/mm^2^ in CTX and an increased OL density of 750.4 ± 82.8 cells/mm^2^ in CC (*p* = 0.0003, *n* = 6 tissue sections). For P60, we captured a tissue area of 8.9 ± 0.6 mm^2^ of CTX and a tissue area of 2.0 ± 0.1 mm^2^ of CC, with an OL density of 170.1 ± 48.2 cells/mm^2^ in CTX and an increased OL density of 608.6 ± 190.4 cells/mm^2^ in CC (*p* = 0.05, *n* = 6 tissue sections), as expected for gray and white matter regions.

**Fig. 3:**
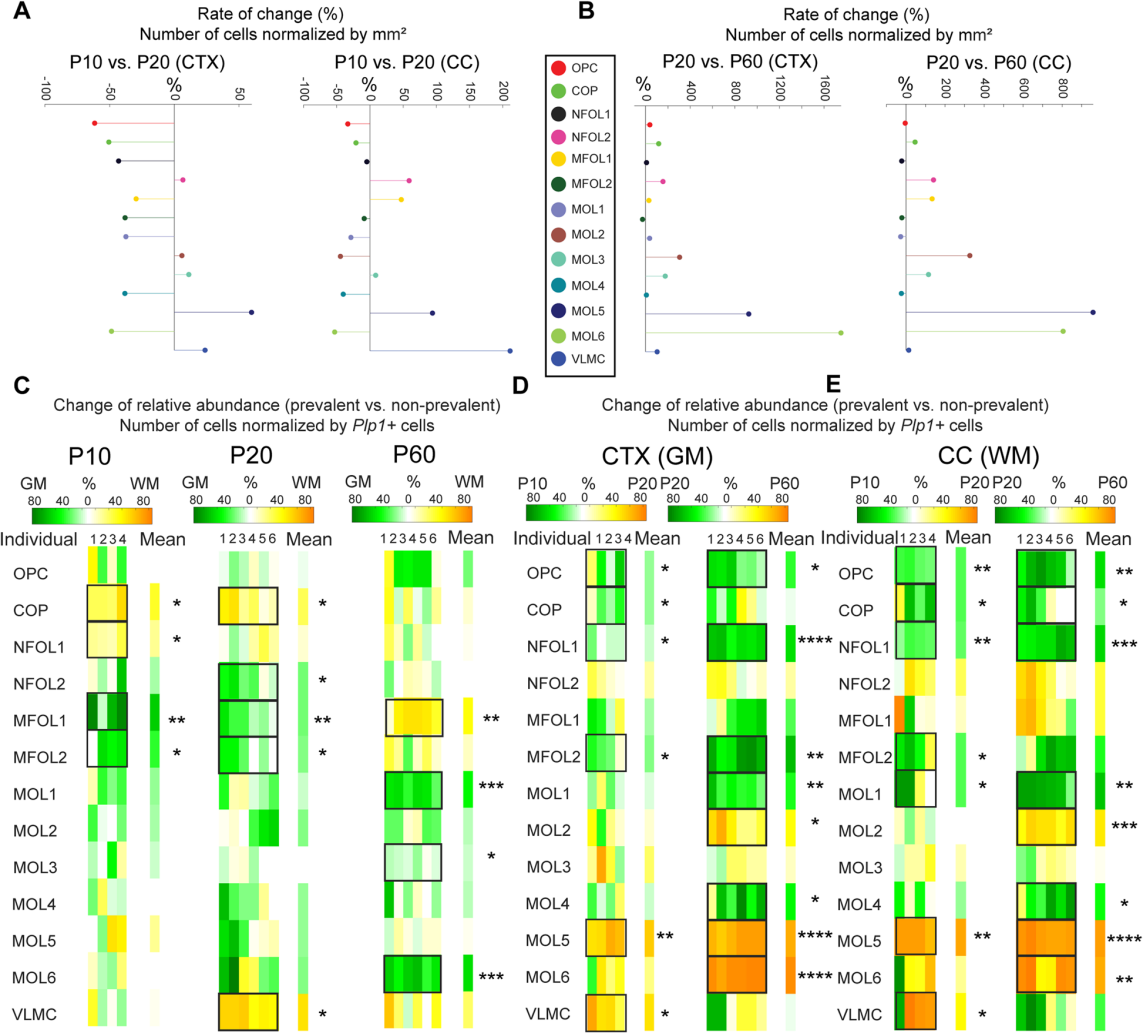
Change of cell abundances per tissue area (cells/mm^2^) and per *Plp1+* cells (%) for P10, P20, and P60 CTX (GM) and CC (WM). **a** Rate of change (%) of cells/mm^2^ in P10 vs. P20 CTX (left) and CC (right). **b** Same as (**a)** for P20 vs. P60 CTX (left) and CC (right). **c** Change of relative abundances of OLs (cells/*Plp1+* cells) between CTX (GM) and CC (WM) for P10 (left), P20 (middle), and P60 (right) (*n* = 4 P10 tissue sections, *n* = 6 P20 tissue sections, *n* = 6 P60 tissue sections). **d** Change of relative abundances of oligodendrocytes between P10 and P20 as well as P20 and P60 for CTX. **e** Same as (**d)** for CC. Statistical comparisons were determined using ANOVA and post hoc test with Tukey correction (*p < 0.05, **p < 0.01, ***p < 0.001, and ****p < 0.0001).

In order to compare the abundances of the OL populations between GM (CTX) and WM (CC) at P10, P20, and P60, we calculated both the cell occurrences per *Plp1*+ cells (%, Fig. 2D–F) and the cell occurrences per tissue area (cells/mm^2^, Additional file 5: Fig. S5A-C). The bar plots of the OL abundances show that at P10 and P20 stages, when myelination is actively ongoing, OPC, COP, NFOL1, and MFOL2 are most abundant in CTX and CC (Fig. 2D, E), while at P60, MOL5 are most abundant in CTX and CC and MOL1 and MOL4 populations are a minority (Fig. 2F). Calculating the rate of change of cells/mm^2^ for each cell population further highlights that the spatial density in P10 CTX is in general higher than in P20 CTX, except for MOL5 and VLMC and some populations that remain stagnant (NFOL2, MOL2, MOL3) (Fig. 3A, left). In P10 CC, the cell densities of NFOL2 and MFOL1 are decreased compared to P20 CC as well as the cell densities of MOL5 and VLMC (Fig. 3A, right). Interestingly, comparing P20 CTX vs. P60 CTX shows that the cell densities of MOL5 and MOL6 strongly increase, while the other densities largely remain stagnant (Fig. 3B, left). Similarly, comparing P20 CC vs. P60 CC shows that the cell densities of MOL5 and MOL6 strongly increase, together with the cell density of MOL2, while the other densities largely remain stagnant (Fig. 3B, right). This could suggest that at the early stages from P10 to P20 (especially in CTX), the decrease in cell density of OPC, COP, NFOL1, and MFOL2 could reflect their differentiation into MOLs, whereas at the later stages, the cell densities for early-stage OLs remain mostly unchanged, which to some extent could reflect their ability to proliferate and to replace those cells that have differentiated.

To further understand the relative changes of OL populations in respect to the overall number of OLs and to compare the abundances across ages and regions, we normalized the number of cells for each OL population by the number of *Plp1*+ cells. We then calculated how much the OL population in the more prevalent region (P10, P20, P60 CTX and CC) is elevated compared to the non-prevalent region. In P10, we found that MFOL1 (59% ± 7% increased in GM versus WM) and MFOL2 (36% ± 12%) are more abundant in GM (CTX) compared to WM (CC), while COP (39% ± 7%) and NFOL1 (22% ± 4%) are increased in WM compared to GM (*p* = 0.009, *p* = 0.01, *p* = 0.03, *p* = 0.04, respectively; *n* = 4 tissue sections) (Fig. 3C, left). In P20, we found that NFOL2 (25 ± 8%), MFOL1 (25 ± 7%), and MFOL2 (22 ± 8%) are more abundant in GM (CTX) compared to WM (CC), while there is an enrichment of COPs (31 ± 8%) and VLMC (56 ± 4%) in CC compared to CTX (*p* = 0.03, *p* = 0.004, *p* = 0.02, *p* = 0.05, *p* = 0.02, respectively; *n* = 6 tissue sections) (Fig. 3C, middle). Interestingly, OPCs were not differentially distributed between these two regions at P10 and P20. At P60, comparing the relative occurrences of OLs over all six tissue sections, we found MFOL1 to be more abundant in WM (CC) compared to GM (CTX) (45 ± 7%), whereas mature OL populations such as MOL1 (45 ± 4%), MOL3 (9 ± 3%), and MOL6 (52 ± 4%) were more enriched in CTX (*p* = 0.004, *p* = 0.0004, *p* = 0.02, *p* = 0.0002, respectively; *n* = 6 tissue sections) (Fig. 3C, right). These data suggest that myelination might be still ongoing in the CC (WM) at P60, while being more advanced in the CTX (GM), since it is more populated with mature OLs. Ongoing oligodendrogenesis and myelination can be observed until late adulthood [9, 10].

### Increased abundance of OPC, COP, NFOL1, and MFOL2 in P10 compared to P20 GM (CTX) and WM (CC)

Comparing the distributions of OL populations between the postnatal and juvenile brain shows an increased abundance of OPC (19 ± 9%), COP (18 ± 10%), NFOL1 (12 ± 5%), and MFOL2 (22 ± 10%) in the P10 CTX compared to P20 CTX (*p* = 0.03, *p* = 0.02, *p* = 0.05, *p* = 0.02, respectively; *n*=4 tissue sections) (Fig. 3D, left). Also, in the CC, OPC (30 ± 2%), COP (11 ± 4%), NFOL1 (28 ± 3%), and MFOL2 (30 ± 10%) as well as MOL1 (29 ± 7%) are increased in P10 compared to P20 (*p* = 0.006, *p* = 0.05, *p* = 0.005, *p* = 0.04, *p* = 0.03, respectively; *n*=4 tissue sections) (Fig. 3E, left). On the contrary, MOL5 (55 ± 4%) and VLMC (57 ± 9%) are increased in the P20 CTX compared to the P10 CTX (*p* = 0.006, *p* = 0.04, respectively; *n*=4 tissue sections) (Fig. 3D, left), and MOL5 (79 ± 3%) and VLMC (47 ± 19%) are also increased in the P20 CC compared to the P10 CC (*p* = 0.01, *p* = 0.04, respectively; *n* = 4 tissue sections) (Fig. 3E, left).

### Decrease of MOL1 and increase of MOL2, MOL5, and MOL6 from P20 to P60 in both GM (CTX) and WM (CC) in the brain

Comparing the distributions of OL populations between the juvenile and adult brain shows a relative decrease in OPC (38 ± 8%), NFOL1 (54 ± 3%), and MFOL2 (64 ± 6%) with age in the CTX (Fig. 3D, right). Interestingly, mature populations as MOL1 (40 ± 6%) and MOL4 (44 ± 15%) were also more abundant at P20 CTX compared to P60 CTX (*p* = 0.02, *p* ≤ 0.0001, *p* = 0.003, *p* = 0.002, *p* = 0.03). MOL1 expresses Egr2/Krox20, which was previously identified as a transcription factor required for the early myelinating phase of Schwann cells [11]. Thus, MOL1 might be an early myelinating population that later transitions into other mature populations. MOL1 is also characterized by the expression of genes involved in response to stress [2, 4], and this reduction might reflect targeted sensitivity or even apoptosis of this OL population. Thus, it is possible that MOL1 represents dying MOLs, which are thus more abundant at earlier stages of postnatal development. In contrast MOL2 (45 ± 9%), MOL5 (77 ± 3%), and MOL6 (87 ± 2%) in CTX were more abundant with age (*p* = 0.01, *p* ≤ 0.0001, *p* ≤ 0.0001, respectively; *n* = 6 tissue sections per age, Fig. 3D, right).

We observed very similar trends in the brain WM (CC), where there was a decrease with age of OPC (50 ± 10%), COP (22 ± 12%), NFOL1 (55 ± 4%), MOL1 (61 ± 8%), and MOL4 (41 ± 16%) and an increase of MOL2 (54 ± 3%), MOL5 (84 ± 1%), and MOL6 (76 ± 8%) (*p* = 0.008, *p* = 0.05, *p* = 0.0005, *p* = 0.004, *p* = 0.03, *p* = 0.0006, *p* ≤ 0.0001, *p* = 0.002, respectively; *n* = 6 tissue sections per age, Fig. 3E, right). Thus, our data suggests that the terminal differentiation into specific mature MOLs occurs at a similar dynamics in GM (CTX) and WM (CC) in the brain (Fig. 3D, E), despite the different pace (Fig. 3C).

### Enrichment of specific OL populations at distinct areas and layers in the CTX

**Fig. 4:**
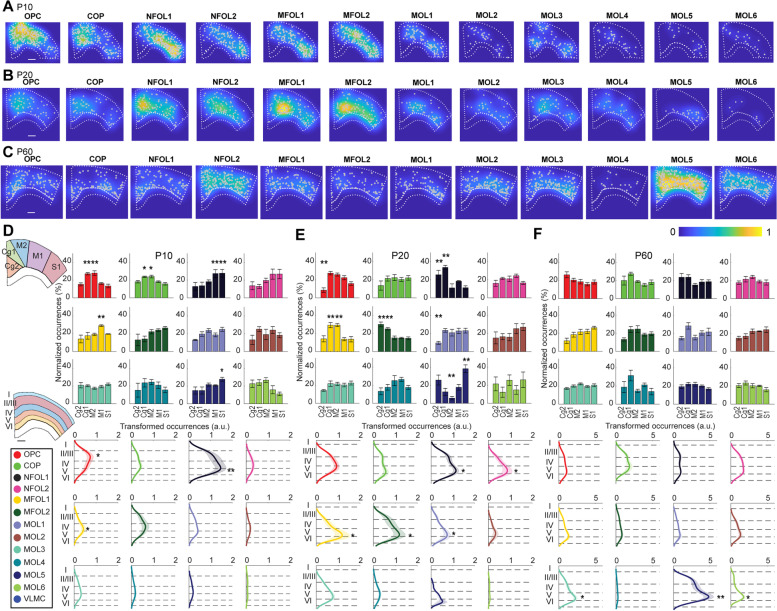
Density of oligodendrocyte types within P10, P20, and P60 CTX (GM). **a** Heatmaps of the density of cells for each oligodendrocyte type in P10 CTX (GM), normalized to the highest cell density per section. The scale bar is 500 μm. **b** Same as (**a)** for P20 CTX. **c** Same as (**a)** for P60 CTX. **d** Top: the mean normalized occurrences of P10 oligodendrocyte populations in primary somatosensory cortex (S1), primary motor cortex (M1), secondary motor cortex (M2), cingulate cortex area1 (Cg1), and cingulate cortex area2 (Cg2) (*n* = 4 P10 tissue sections). Bottom: the mean cortical depth profiles with the transparent shades representing the standard error of the mean. Dashed lines denote layers I, II/III, IV, V, and VI. The *X*-axis (top) and *Y*-axis (bottom) respectively represent the occurrences after smoothening non-binned data with a kernel statistical comparisons were determined using ANOVA and post hoc test with Tukey correction (*p < 0.05, **p < 0.01, ***p < 0.001, and ****p < 0.0001). **e** Same as (**d)** for P20 CTX (*n* = 6 P20 tissue sections). **f** Same as (**d)** for P60 CTX (*n* = 6 P60 tissue sections)

Specific populations of neurons and even fine transcriptomic cell types are differentially distributed in six distinct layers and areas in the CTX [12, 13]. We thus investigated whether the same applies to OLs. First, we plotted the kernel density estimates for each of the twelve OL populations on top of the cell maps, highlighting the spatial preference of the OL populations (Fig. 4A–C). The heatmaps are normalized to the highest cell density per section and reflect the relative abundances; cell maps of raw data are plotted in Additional file 3: Fig. S3. We further segmented the sections according to the Allen Brain Atlas into the primary somatosensory cortex (S1), primary motor cortex (M1), secondary motor cortex (M2), cingulate cortex area1 (Cg1), and cingulate cortex area2 (Cg2) [14].

At P10, we found OPC and COP being more abundant in the medial regions M2 and Cg1, while in the lateral regions, NFOL1 (M1 and S1), MFOL1 (M1), and MOL5 (S1) are being more abundant (OPC: *p* = 0.005, *p* = 0.004; COP: *p* = 0.02, *p*=0.02; NFOL1: *p* = 0.007, *p* = 0.007; MFOL1: *p* = 0.007; MOL5: *p* = 0.04, respectively; *n* = 4 tissue sections per condition, Fig. 4D, top). We further segmented the five neocortical layers and found that OPC exposed an increased occurrence in the supragranular layers and NFOL1 and MFOL1 showed an increased occurrence in the infragranular layers (*p* = 0.02, *p* = 0.007, *p* = 0.04, respectively; *n*= 4 tissue sections per condition, Fig. 4D, bottom).

At P20, we observed a clear transition of early myelinating populations from lateral towards medial regions of the CTX. We found NFOL1, MFOL1, and MFOL2 being more abundant in M2, Cg1, and Cg2. OPC and MOL1 presented a similar distribution in P20 as P10 being decreased in Cg2 (OPC: *p* = 0.004; NFOL1: *p* = 0.003, *p* = 0.002; MFOL1: *p* = 0.005, *p* = 0.005; MFOL2: *p* = 0.005, *p* = 0.008; MOL1: *p* = 0.002; MOL5: *p* = 0.002, *p* = 0.003, respectively; *n* = 6 tissue sections per condition, Fig. 4E, top). Mature OL populations were slightly more abundant, and equally distributed in the different areas, with the exception of MOL5, which was more prevalent in S1 than in other regions and less prevalent in M2 than in other regions (*p* = 0.002, *p* = 0.003, respectively). Thus, our ISS analysis indicates that the different areas in the CTX are in different stages of OL lineage progression in the postnatal and juvenile brain.

The layer analysis in P20 also showed that there are fewer OLs in layer I compared to deeper layers (I vs. II/III: *p* = 0.001; I vs. IV: *p* = 0.0008; I vs. V: *p* = 0.0006; I vs. VI: *p* = 0.0003, *n* = 6 tissue sections per condition), in accordance with the literature [15, 16]. In addition, there were less OLs in layer II/III and layer IV compared to layer V and layer VI, respectively (II/III vs. V: *p* = 0.001; II/III vs. VI: *p* = 0.004; IV vs. V: *p* = 0.03; IV vs. VI: *p* = 0.05, *n* = 6 tissue sections per condition). OLs associated with early stages of differentiation and myelination, such as NFOL1, NFOL2, MFOL1, and MFOL2 in P20 brains exposed an increased occurrence in the infragranular layers, peaking at layer V and VI (*p* = 0.01, *p* = 0.01, *p* = 0.02, *p* = 0.03, *p* = 0.03, respectively; *n* = 6 tissue sections per condition, Fig. 4E, bottom). These data could suggest that the cellular environment at layers V and VI might actively be involved with the induction of OL differentiation and myelination. Interestingly, MOL1 was also enriched in this layer at P20 (*p* = 0.03; *n* = 6 tissue sections, Fig. 4E, bottom). OL differentiation and OL stress/apoptosis are closely connected [17], and our data suggests that the local environment at the cortical layer V at P20 might be amenable for both these processes.

There is a substantial increase in the numbers of MOLs from P20 to P60. However, in contrast to P10 and P20, we did not observe any enrichment in the different cortical areas at P60 (Fig. 4F, top), suggesting that the process of differentiation, myelination, and subtype specification is very advanced at this stage (although it is possible that specified MOL can still form new myelin sheaths later in late adulthood). Given that OPC and COP are being more abundant in the medial regions M2 and Cg1 at P10, while NFOL1 (M1 and S1), MFOL1 (M1), and MOL5 (S1) are more abundant in the lateral regions (Fig. 4A), a burst of differentiation and myelination might occur prior to P20 in the lateral regions. These early differences in oligodendrocyte lineage progression between the lateral and medial regions might be compensated later on within the medial regions after P20, to achieve a homogenous distribution of MOLs at P60.

Similar to P20, there were less OLs in layer I compared to deeper layers (I vs. II/III: *p* = 0.02; I vs. IV: *p* = 0.02; I vs. V: *p* = 0.001; I vs. VI: *p* = 0.001, *n* = 6 tissue sections per condition) as well as less OLs in layer II/III and layer IV compared to layer V and layer VI, respectively (II/III vs. V: *p* = 0.03; II/III vs. VI: *p* = 0.02; IV vs. V: *p* = 0.05; IV vs. VI: *p* = 0.05, *n* = 6 tissue sections per condition). In P60 cortical brain sections, the early differentiating/myelinating OLs and MOL1 presented a more uniform distribution when compared to P20. Moreover, there was an increased abundance in deeper regions, particularly again at layer V/VI, of mature OL populations such as MOL3, MOL5, and MOL6 populations (*p* = 0.05, *p* = 0.008, *p* = 0.04, respectively; *n* = 6 tissue sections, Fig. 4E, bottom). Thus, our data suggest that layers V and VI are particularly enriched for myelinating OL at P20, especially in the medial regions, while at P60, these layers are enriched in specific mature OL populations.

### Differential dynamics and abundances in brain and spinal cord WM and GM

**Fig. 5:**
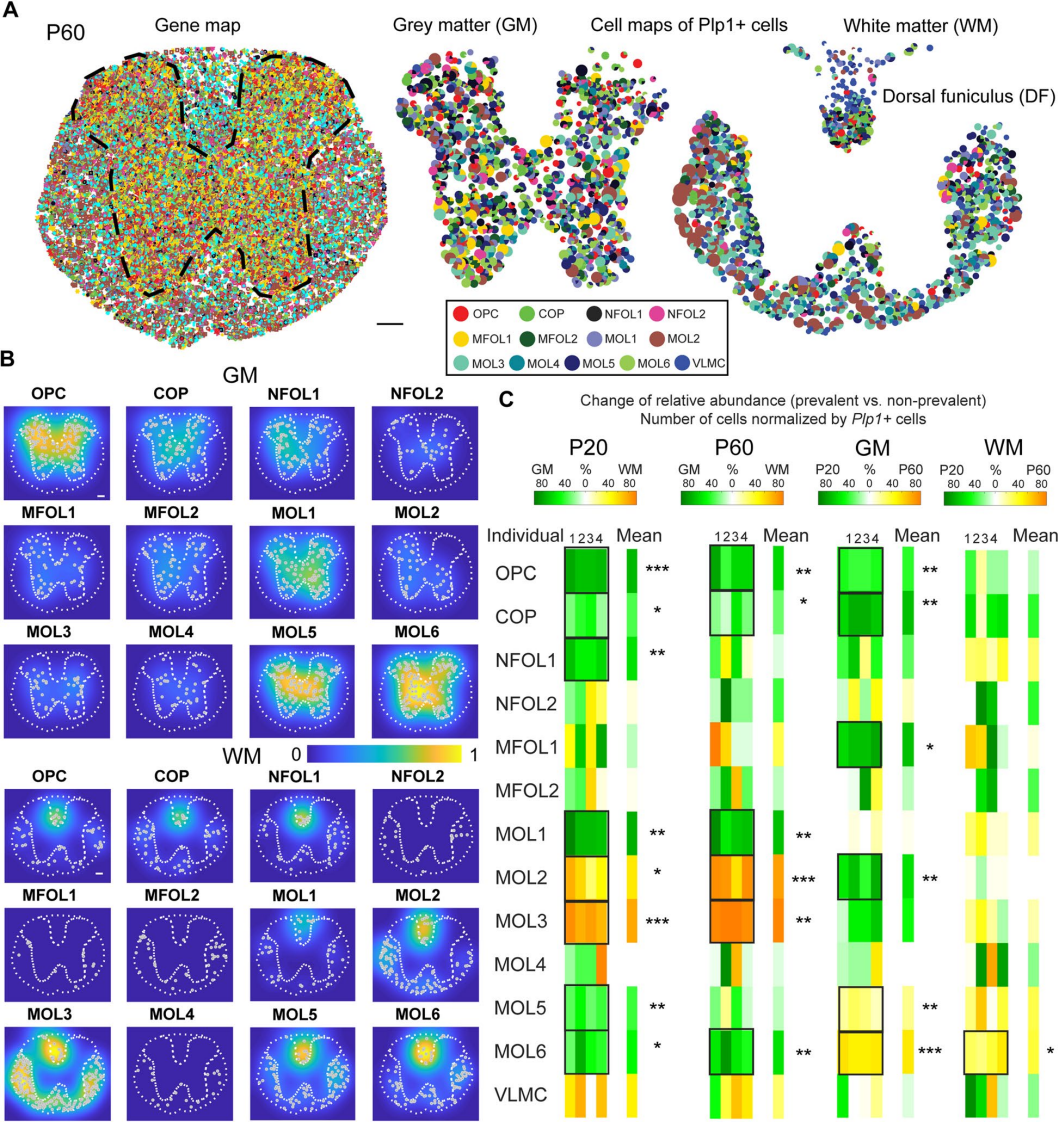
Oligodendrocyte types in spinal cord gray matter (GM) and white matter (WM). **a** Maps of 124 genes (left) and 13 cell types (middle and right) in the spinal cord sections (P60) targeted by in situ sequencing. The dashed line in the gene map highlights the separation between gray matter (GM) and white matter (WM). The scale bar is 100 μm. **b** Heatmaps of the density of cells for each oligodendrocyte type, normalized by the maximum density over all types. The scale bar is 100 μm. **c** Change of relative abundances of oligodendrocytes between GM and WM for P20 and P60 (left) as well as change of relative abundances of oligodendrocytes between P20 and P60 for GM and WM (right) (*n* = 6 P20 tissue sections, *n* = 6 P60 tissue sections). Statistical comparisons were determined using ANOVA and post hoc test with Tukey correction (*p < 0.05, **p < 0.01, ***p < 0.001, and ****p < 0.0001)

Next, we investigated the occurrence of the OL populations in the P20 and P60 spinal cord. In total, we captured a tissue area of 1.1 ± 0.2 mm^2^ of P20 GM and a tissue area of 1.0 ± 0.1 mm^2^ of P20 WM. For P60, we captured a tissue area of 1.4 ± 0.2 mm^2^ of GM and a tissue area of 1.2 ± 0.2 mm^2^ of WM. The gene map of a thoracic P60 spinal cord section is shown in Fig. 5A, left, with colored symbols representing the unique transcripts, and the segmented gray matter (GM) and white matter (WM) cell maps are shown in Fig. 5A, right. Similar to the brain, we also plot kernel density estimates for each of the twelve OL populations on top of the cell maps, highlighting the spatial preference of the OL populations (Fig. 5B). Note that we split the heatmaps for GM and WM to allow direct comparisons and highlight the cellular densities in the dorsal funiculus region of WM. The cell maps of raw data are shown in Additional file 6: Fig. S6.

Comparing the relative cell occurrences (in respect to the overall number of Plp1+ cells) showed on average an enrichment of OPC (72 ± 2%), COP (33 ± 5%), NFOL1 (59 ± 3%), MOL1 (76 ± 4%), MOL5 (36 ± 4%), and MOL6 (48 ± 9%) in P20 GM compared to P20 WM in the spinal cord. In contrast, MOL2 (54 ± 7%) and MOL3 (79 ± 7%) were enriched in P20 WM (*p* = 0.0003, *p* = 0.01, *p* = 0.002, *p* = 0.002, *p* = 0.003, *p* = 0.02, *p* = 0.02, *p* = 0.0009, respectively; *n* = 6 tissue sections per condition, Fig. 5C, left). These distributions were not substantially altered in P60 spinal cord, with an average enrichment of OPCs (60 ± 7%), COP (29 ± 7%), MOL1 (70 ± 6%), and MOL6 (59 ± 8%) in GM compared to WM and reversibly an average enrichment of MOL2 (80 ± 6%) and MOL3 (92 ± 1%) in WM compared to GM (*p* = 0.002, *p* = 0.04, *p* = 0.003, *p* = 0.007, *p* = 0.005, *p* = 0.002, respectively; *n* = 6 tissue sections per condition, Fig. 5C, left). MOL2/3 and MOL5/6 are also densely present in the dorsal funiculi of WM (Fig. 5B). Thus, in contrast to the brain, we did not observe major differences in the pace of differentiation/myelination between P20 and P60. Nevertheless, there is a clear spatial preference for MOL2 in WM and MOL5/6 in GM at P20 and P60, as we have previously reported [4].

While there is a decrease with age in the GM of OPC (39 ± 2%), COP (69 ± 3%), MFOL1 (70 ± 3%), and MOL2 (60 ± 6%), there is an increase of MOL5 (23 ± 3%) and MOL6 (54 ± 1%) (*p* = 0.002, *p* = 0.001, *p* = 0.03, *p* = 0.008, *p* = 0.008, *p* = 0.00013, respectively; *n* = 6 tissue sections per condition, Fig. 5C, right). In the WM, a similar pattern was observed for MOL6 (37 ± 7% increase). However, in contrast to GM, OPC, COP, MFOL1, MOL2, and MOL5 (*p* = 0.06; *n* = 6 tissue sections per condition) were not significantly altered in WM (Fig. 5C, right). Also, in contrast to the brain, MOL1 was not reduced from P20 to P60 in the spinal cord, suggesting a regional specificity in the dynamics and abundance of OL populations in the WM and GM of different regions of the CNS.

**Fig. 6:**
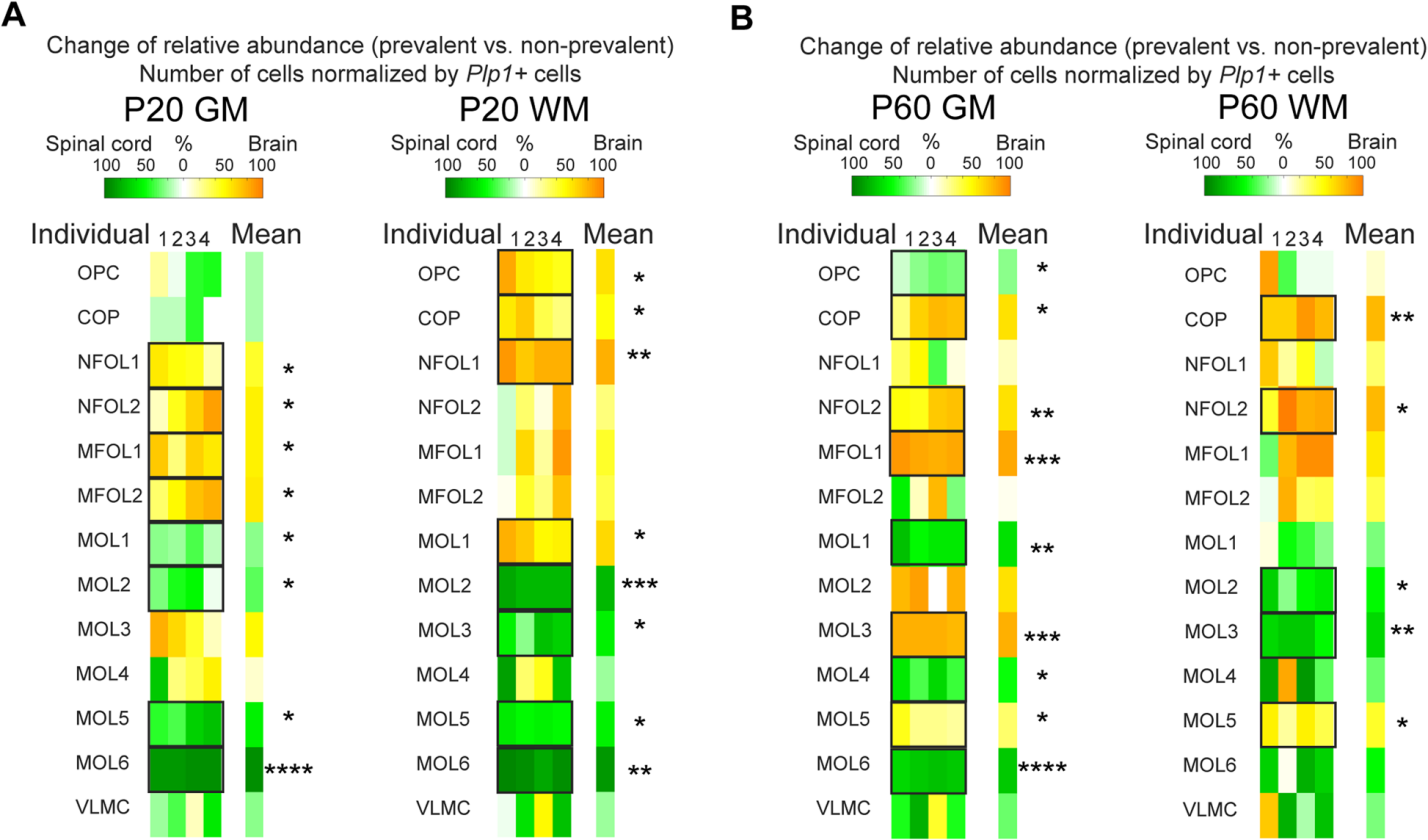
Change of relative oligodendrocyte abundances (cells/*Plp1+* cells) in P20 and P60 GM and WM for brain vs. spinal cord. **a** Change of relative abundances of oligodendrocytes between the brain and spinal cord for P20 GM (left) and P20 WM (right) (*n* = 6 P20 tissue sections, *n* = 6 P60 tissue sections). Statistical comparisons were determined using ANOVA and post hoc test with Tukey correction (*p < 0.05, **p < 0.01, ***p < 0.001, and ****p < 0.0001). **b** Change of relative abundances of oligodendrocytes between the brain and the spinal cord for P60 GM (left) and P20 WM (right).

Comparing the relative OL abundances of brain vs. spinal cord showed for P20 GM increases for NFOL1 (42 ± 7%), NFOL2 (53 ± 13%), MFOL1 (55 ± 8%), and MFOL2 (58 ± 9%) in the brain and increases of MOL1 (24 ± 4%), MOL2 (33 ± 9%), MOL5 (54 ± 8%), and MOL6 (91 ± 1%) in the spinal cord (*p* = 0.02, *p* = 0.05, *p* = 0.04, *p* = 0.05, *p* = 0.01, *p* = 0.05, *p* = 0.03, *p* = 0.00003, respectively; *n* = 6 tissue sections per condition, Fig. 6A, left), indicating that the GM in the spinal cord is in a more advanced maturation state than in the brain. A similar pattern is found in the WM, where at P20, there is an increase of OPC (60 ± 7%), COP (48 ± 8%), NFOL1 (79 ± 3%), and MOL1 (64 ± 6%) in the brain and an increase of MOL2 (78 ± 1%), MOL3 (54 ± 9%), MOL5 (55 ± 1%), and MOL6 (90 ± 2%) in the spinal cord (*p* = 0.04, *p* = 0.05, *p* = 0.002, *p* = 0.01, *p* = 0.0005, *p* = 0.02, *p* = 0.006, *p* = 0.01, respectively; *n* = 6 tissue sections per condition, Fig. 6A, right).

At P60, GM showed increases of COP (61 ± 10%), NFOL2 (60 ± 6%), MFOL1 (83 ± 2%), and MOL3 (79 ± 1%) in the brain and increases of OPC (22 ± 3%), MOL1 (60 ± 4%), MOL4 (49 ± 5%), and MOL6 (70 ± 2%) in the spinal cord (*p* = 0.03, *p* = 0.006, *p* = 0.0003, *p* = 0.0003, *p* = 0.02, *p* = 0.001, *p* = 0.03, *p* = 0.00001, respectively; *n* = 6 tissue sections per condition, Fig. 6B, left). P60 WM shows increases of COP (75 ± 4%), NFOL2 (75 ± 10%), and MOL5 (41 ± 7%) in the brain and increases of MOL2 (50 ± 8%) and MOL3 (63 ± 4%) in the spinal cord (*p* = 0.004, *p* = 0.02, *p* = 0.02, *p* = 0.03, *p* = 0.007, respectively; *n* = 6 tissue sections per condition, Fig. 6B, right). Interestingly, at P60, MOL2 is enriched in the WM spinal cord versus the WM brain, but the opposite seems to apply to the GM. MOL1 and MOL6 appear more enriched in the spinal cord, while MOL5 in the brain at P60. Thus, at P60, region-specific differences on the abundance of the mature OL populations become clearer and consolidated.

### Oligodendroglia populations’ neighboring preferences are altered from the juvenile to the adult CNS

**Fig. 7:**
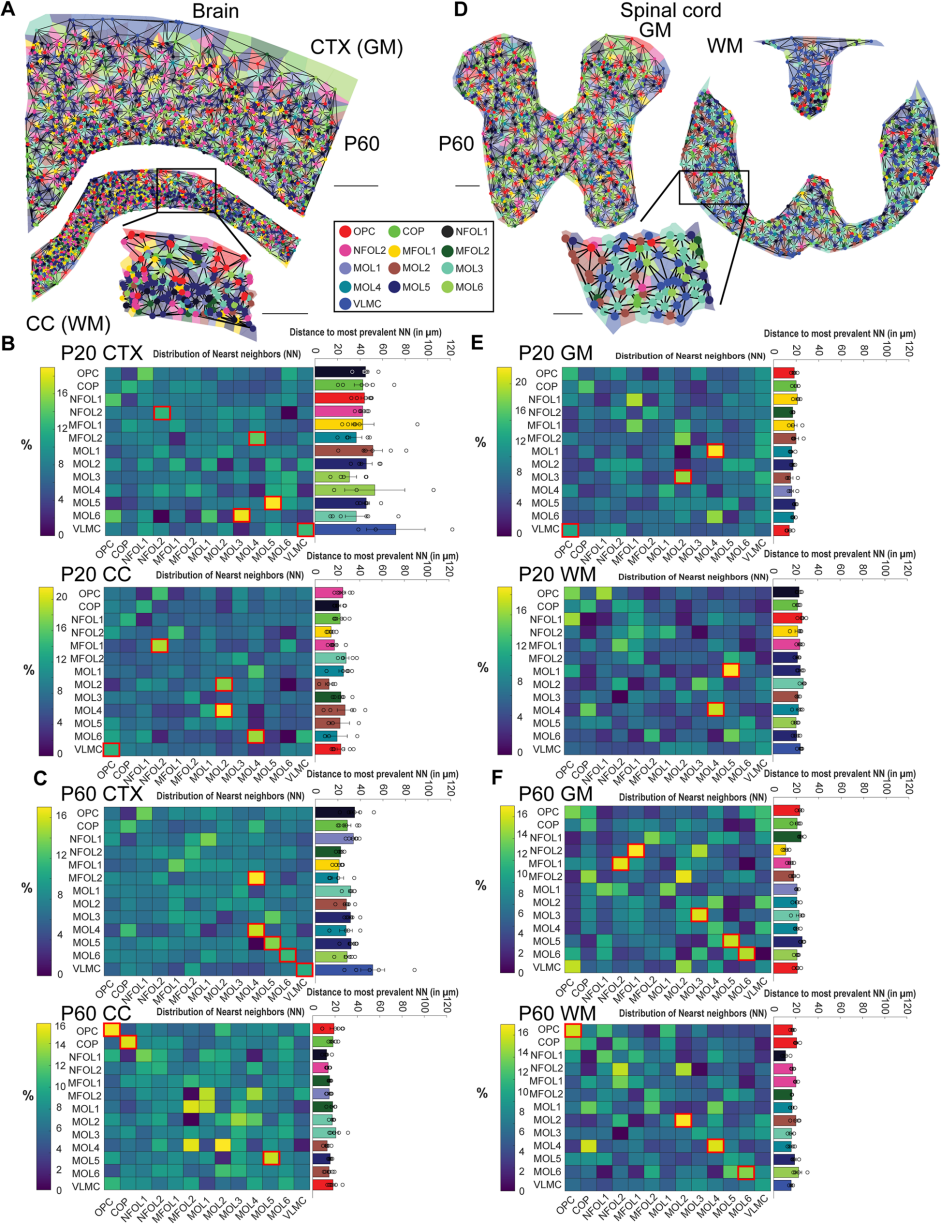
Nearest neighbor (NN) analysis for oligodendrocytes in P20 and P60 tissue sections. **a** For each cell, a region is plotted (tiles in the background), where all points of the region are closer to that cell than to any other (Voronoi tessellation). Each cell is a node in the graph and the edges connect the neighboring cells (Delaunay triangulation). A zoom-in is shown for the WM region. The main scale bar is 500 μm, and the zoom-in scale bar is 250 μm. **b** Heatmaps of the nearest neighbor likelihood (significant neighboring preferences highlighted by red boxes) and barplot of average distances to most prevalent NN for P20 CTX (top) and CC (bottom) (*n* = 6 P20 tissue sections). **c** Same as (**b)** for P60 CTX and CC (*n* = 6 P60 tissue sections). **d** Same as (**a)** for the spinal cord. The main scale bar is 100 μm, and the zoom-in scale bar is 50 μm. **e** Same as (**b)** for spinal cord P20 GM and WM (*n* = 6 P20 tissue sections). **f** Same as (**e)** for spinal cord P60 GM and WM (*n* = 6 P60 tissue sections)

As OL populations show different spatial preferences in the brain and spinal cord, the question arises if certain OL populations are organized in distinct neighborhoods. Therefore, we performed a nearest neighbor analysis, where each cell is represented as a node in a graph. For each node, there is a corresponding region consisting of all points of the plane closer to that node than to any other. This mathematical approach partitions the plane into regions and is called Voronoi tessellation (see shades in Fig. 7A, D). Each node is then connected to all its neighboring nodes (Delaunay triangulation), and the distance between the points can be measured using the Euclidean distance. The neighbor with the closest Euclidean distance is the nearest neighbor. We calculated the nearest neighbor distances for each OL population for P20 GM and WM as well as P60 GM and WM and visualized the distribution of relative abundances of the nearest neighbors in heatmaps (Fig. 7B–F). Therefore, the absolute abundances of the nearest neighbors were normalized for each cell type, i.e., the relative abundances represent the percentages of instances in which a certain cell type is the nearest neighbor.

To assess the arrangement of the OLs, we applied neighborhood enrichment analysis similar to [18]. This analysis utilizes a permutation-based test and compares the cell type labels with a random configuration of labels by maintaining the positional information. In the P20 CTX, we found that NFOL2 had the highest neighboring preference with NFOL2, MFOL2 with MOL4, MOL5 with MOL5, and MOL6 with MOL3 (*p* = 0.05, *p* = 0.04, *p* = 0.01, *p* = 0.01, respectively; *n* = 6) (Fig. 7B, top, red boxes). These neighboring preferences were statistically different from the randomized positioning in the simulated data. In the P20 CC, we found the neighboring of MFOL1 with NFOL2, MOL2 and MOL4 with MOL2, and MOL6 with MOL4 significantly different (*p* = 0.04, *p* = 0.05, *p* = 0.006, *p* = 0.03, respectively; *n* = 6) (Fig. 7B, bottom, red boxes). The average Euclidean distances between OL populations, however, were not significantly different in the P20 CTX, being on average between 40 and 50 μm. In the P20 CC, the distances were even more variable, ranging from 15 μm for MOL2-MOL2 to nearly 30 μm for MFOL2-MOL3.

Interestingly, in the P60 CTX, we found MOL4 preferentially neighboring MOL4, MOL5 preferentially neighboring MOL5, MOL6 preferentially neighboring MOL6, and MFOL2 neighboring with MOL4 (*p* = 0.004, *p* = 0.02, *p* = 0.05, *p* = 0.03, respectively; *n* = 6) (Fig. 7C, top, red boxes). In the P60 CC, we found the neighboring of OPC with OPC, COP with COP, and MOL5 with MOL5 significant (*p* = 0.04, *p* = 0.05, *p* = 0.006, *p* = 0.03, respectively; *n* = 6) (Fig. 7C, bottom, red boxes), suggesting a homotypic neighborhood structure with some OLs preferentially neighboring with OLs of the same type. The distances between OL populations in P20 CTX ranged from 20 to 40 μm, and in P60 CC, from 10 to 25 μm. Interestingly, the homotypic neighborhood structure is also visible in the spinal cord, especially at P60 (Fig. 7F).

While in P20 we only found MOL4 preferentially neighboring MOL4 in WM (*p* = 0.04; *n* = 6) in addition to some heterotypic neighboring (MOL1 with MOL4 in GM: *p* = 0.001, MOL3 with MOL2 in GM: *p* = 0.05, MOL1 with MOL5 in WM: *p* = 0.003; *n* = 6) (Fig. 7E, red boxes), we found striking patterns in P60 GM and P60 WM. In the P60 GM, we found NFOL2 preferentially neighboring with MFOL1 and vice versa, MOL3 with MOL3, MOL5 with MOL5, and MOL6 with MOL6 (*p* = 0.04, *p* = 0.0008, *p* = 0.03, *p* = 0.04, *p* = 0.03, respectively; *n* = 6) (Fig. 7F, top, red boxes). In the P60 WM, we found the neighboring of OPC with OPC, MOL2 with MOL2, MOL4 with MOL4, and MOL6 with MOL6 significant (*p* = 0.04, *p* = 0.02, *p* = 0.05, *p* = 0.05, respectively; *n* = 6) (Fig. 7F, bottom, red boxes). This homotypic neighborhood structure could reflect a stabilization of the OL phenotypes in adulthood, due to a reduction of migration of OPCs and stable myelin wrapping of defined neuronal populations in the adult brain.

Interestingly, VLMCs mainly show preference to other VLMCs or to OPCs (P20 CTX VLMC-VLMC: *p* = 0.04, P20 CC VLMC-OPC: *p* = 0.01, P60 CTX VLMC-VLMC: *p* = 0.02, P20 GM VLMC-OPC: *p* = 0.05; *n* = 6) (Fig. 7B–F). VLMCs are associated with the vasculature and can thereby be a proxy for vasculature proximity at the CNS parenchyma. OPCs have been reported to be closely associated with the vasculature during embryonic development and during their migration [19]. While OPC migration in the adult CNS is limited, it is reactivated upon injury, alongside OPC association with the vasculature [20]. Thus, our nearest neighbor analysis supports the association of OPCs with vasculature.

## Discussion

In this study, we analyzed simultaneously the expression of 124 genes expressed in different OL lineage populations in three regions of the juvenile and adult CNS. In situ sequencing (ISS) and pciSeq analysis allowed us to determine the spatial distribution of OLs with an unprecedented resolution. The pciSeq cell calling algorithm yields probabilistic readouts based on prior scRNA-seq classification. The algorithm performs well on different scRNA-seq datasets, e.g., in our previous study [21], we have successfully applied pciSeq on human cortical tissue sections, leveraging two independent scRNA-seq datasets. However, since oligodendrocytes are more in a developmental continuum compared to neurons and to minimize the risk of potential false classification, we only considered cells expressing *Plp1* for our analysis, as *Plp1* expression is particularly high in OL lineage cells [2] (see Fig. 1C).

In the present study, the distribution of OL lineage cells in the forebrain WM and GM at P10, P20, and P60 suggests that differentiation and myelination advance earlier in CTX (GM) than in CC (WM) in the brain. We observed an enrichment of COPs in WM at P10 as well as at P20, while more intermediate stages such as NFOL2 and MFOL1/2 were enriched in the GM (Fig. 3A). This data suggest that differentiation is actively ongoing at this stage in the CC, while in CTX, OLs are rather in a myelination phase. Indeed, using lineage tracing with the NG2creERTMBAC:ZEG mice, Zhu and colleagues showed that OL differentiation plateaus between P10 and P60 in the CTX, while it is still occurring until P60 in the CC [22]. Interestingly, these intermediate myelin-forming states are enriched in the CC WM at P60, while the CTX GM has already transitioned to fully mature OLs (Fig. 3A). This is consistent with previous lineage tracing findings with various transgenic mouse lines, indicating that OPCs in the cortical GM have a longer cell cycle time than in WM CC at P20 and P60 [23, 24] and that differentiation and myelination of the CC WM can extend until at least 240 days in the mouse, but to a lesser extent in the CTX GM [22, 25–27]. An interesting aspect here would be to include more cell cycle/proliferation markers into future probe panel designs to further elucidate the spatial differences between OPCs and MOLs in GM and WM in terms of cell cycle time/proliferation and differentiation rates as shown by [23].

In the P60 WM and GM in the brain, the composition of mature OL populations is quite similar, with a marked increase in MOL6 and MOL5, which become the prevalent populations in these regions (Figs. 2 and 3). In the spinal cord, the spatial preferences of oligodendroglial populations are even more pronounced, which are maintained from P20 to P60 (enrichment of OPC/COP, MOL1, and 5/6 in GM and MOL2/3 in WM). Here, it would be interesting to further investigate whether such clear spatial preference is also established later on in adulthood in the brain. Alternatively, it is possible that other regions in the brain (for instance, in ventral regions) exhibit more pronounced spatial preferences of certain OL populations.

In this study, we observed a decrease in MOL1 consistent with our previous RNASCOPE dataset, where there was a tendency for a decrease in the MOL1 population with age [4], which becomes clear with ISS. MOL1 is a population we previously observed to be dramatically increased at the injury site upon spinal cord injury [4]. The observed developmental decrease in MOL1 occurs as populations such as NFOLs, and MFOLs are also reduced, not only as a result of the dynamics of differentiation between the two time points, but also due to programmed cell death [17, 28–30]. The presence of MOL1 in niches during development and pathology that are associated with cell death could suggest that this mature OL state might be associated with stress and even apoptosis. Indeed, MOL1 is characterized by the expression of genes such as *Egr1*, *Egr2*, *Fos*, and *Arc*, among others [2]. Many of these genes can be upregulated upon neuronal activation but also upon stress and apoptosis [31]. However, stress and apoptosis can be induced by the processing of tissue in single-cell transcriptomic approaches [32, 33]. Thus, it is challenging to determine whether stress/activated related signatures are truly biological or artifactual. Nevertheless, the detection of MOL1 in situ in the CNS by technologies such as RNASCOPE [4] and ISS (this study) strongly suggests that this population represents a physiological state of mature oligodendrocytes, although it remains unclear if it is a stress-related transient state, which might become stabilized in certain developmental or pathological niches.

One of the major advantages of technologies such as ISS is the spatial resolution, and the possibility to examine population distributions in anatomical areas. We, therefore, superimposed our data with previously defined cortical areas. Interestingly, at P10, we observed newly formed (NFOL1) and myelin-forming (MFOL1) oligodendrocytes present mainly in the deep layers of the somatosensory cortex and motor cortex 1, while at P20, we observed a higher abundance in the cingulate and motor cortex 2 areas. At P60, we observed a rather uniform distribution of the OL lineage populations. Our data, thus, indicates a lateral-to-medial gradient of OL lineage progression in the CTX and possibly a deep-to-superficial layer gradient. While most of the mature OL lineages have been previously shown to accumulate in the deeper layers of the cortex [9, 15, 16], the lateral-to-medial gradient was unexpected and suggests that OL differentiation and myelination occur at different time points in different areas in the cortex. Such arealization of myelination has been shown in the mouse corpus callosum between P3 and P21, with sensory pathways being myelinated first, followed by motor pathways and association pathways [16], and also in humans [34, 35]. Interestingly, specific mature OL populations have been shown to myelinate inhibitory neurons, excitatory neurons, or both [36], although it is unclear if these populations have any correspondence to the transcriptional states as MOL1–6. It is possible that neurons and other cells such as microglia present in layers V–VII might have a particular role in the differentiation, myelination, and final subtype specification of MOLs. Neuropilin-1 has been recently shown to regulate OPC proliferation and to be expressed only in activated microglia in the developing WM (CC), but not in GM (CTX) or adult CC [37]. It is also possible that distal neuronal connections modulate the timing of OL lineage progression in the cortex, as many of these cortical areas project to distinct and remote areas of the CNS.

We also observed that the maturation into MOLs is more advanced at P20 in both WM and GM of the spinal cord, when compared to the brain (Fig. 6). This is consistent with previous reports of an posterior-to-anterior gradient of myelin glycoprotein (Mog) expression in the post-natal rat CNS [38], autopsy studies in humans [35], longer cell cycle time of OPCs in the P21 and P60 spinal cord WM compared to the corpus callosum [23], and reduced capacity of differentiation of OPCs at the P30 in the spinal cord when compared to the brain [22, 25–27].

In our previous study, based on RNASCOPE, MOL2 cells could barely be detected in CTX and CC and were prominent in the spinal cord [4]. Using several markers for MOL2, we could confirm the prevalence of this population in the spinal cord WM versus brain WM (Fig. 6B), but could now detect a higher number of this population in the brain and an increase in these brain regions from P20 to P60 (Fig. 3B). This suggests that MOL2 might also have relevant functions not only in the spinal cord, but also in the brain, which could be particularly relevant given that we observed that MOL2 is particularly affected in the context of injury [4].

Another important aspect to consider is that our neighborhood analysis is centered on the nucleus of each cell. OPCs and OLs exhibit extensive processes, which sense the environment and neighboring cells. Thus, while the cell body of certain cells might be distant, it is possible that their processes are in close contact. Interestingly, our nearest neighbor analysis indicates that the closest neighbors of OPCs are VLMCs or OPCs themselves (Fig. 7). However, OPCs have been shown to self-avoid in the mouse adult (P60–P90) somatosensory CTX, thereby maintaining distinct territories [39]. This apparent paradox most likely reflects the high level of area coverage of OPCs in the CNS, as observed in Figs. 4 and 5. Cell death and differentiation of OPCs are compensated by proliferation and migration of neighboring OPCs, maintaining thus a constant and high density [39, 40] and remodeling [41], and response to inflammatory insults in the context of injury or disease [42].

## Conclusions

Our ISS dataset provides the first detailed analysis of oligodendrocyte lineage spatial organization in the mouse juvenile and adult WM and GM. While confirming previous literature findings regarding the caudal-rostral timing of myelination and association of OPCs with the vasculature, our data extends these findings by giving a detailed view of the spatial organization of distinct OL lineage populations. Furthermore, it uncovers the timing of OL differentiation and myelination in specific areas of the cortical GM and OL lineage neighborhoods. Future integration of our study with other emerging spatial transcriptomics investigations, such as targeting in detail subtypes of neurons, astrocytes, and microglia, among others, is bound to lead to a better understanding of the cellular architecture of the CNS and the principles by which it is established.

## Methods

### Animals

All animals (P10: Sox10-Cre/RCE, P20 and P60: Pdgfrα::CreERTM-loxP-RCE (Z/EG) [27] with mixed C57BL/6NJ and CD1 background) were free from the most common mouse viral pathogens, ectoparasites, endoparasites, and mouse bacterial pathogens harbored in research animals. The battery of screened infective agents met the standard health profile established in Karolinska Institutet animal housing facilities. Mice were kept with the following light/dark cycle: dawn 6:00–7:00, daylight 7:00–18:00, dusk 18:00–19:00, and night 19:00–6:00, and housed to a maximum number of five per cage in individually ventilated cages (IVC Sealsafe GM500, Tecniplast). Cages contained hardwood bedding (TAPVEI), nesting material, shredded paper, gnawing sticks, and a cardboard box shelter (Scanbur). The mice received a regular chew diet (either R70 diet or R34, Lantmännen Lantbruk, or CRM-P, 801722, Special Diet Services). General housing parameters such as relative humidity, temperature, and ventilation follow the European convention for the protection of vertebrate animals used for experimental and other scientific purposes treaty ETS 123. Briefly, consistent relative air humidity of 50%, 22 °C, and the air quality is controlled with the use of stand-alone air handling units supplemented with high-efficiency particulate air-filtered air. Husbandry parameters were monitored using ScanClime (Scanbur) units. Water was provided in a water bottle, which was changed weekly. Cages were changed every other week. All cage changes were done in a laminar airflow cabinet. Facility personnel wore dedicated scrubs, socks, and shoes. Respiratory masks were used when working outside of the laminar airflow cabinet.

### In situ sequencing assay

P10, P20, and P60 mouse brain tissues and spinal cord tissues were snap-frozen in OCT mounting medium, coronally sectioned in 10-μm-thick cryosections, and stored at − 80 °C until fixation. Sixteen coronal brain sections (bregma 1.10 mm) and 8 thoracic spinal cord sections were studied. The brain sections were hemisected. In situ sequencing (ISS), a targeted multiplexed mRNA detection assay employing padlock probes, rolling circle amplification (RCA), and barcode sequencing, was applied. Padlock probes targeting a set of 124 genes and the CartaNA NeuroKit (1010-01, CartaNA AB, now part of 10x Genomics) were used (similar to [43]). For a step-by-step protocol of the in situ sequencing chemistry, see [44]. In short, 10-μm-thick cryosections were fixed by 3% (w/v) paraformaldehyde (PFA) in DEPC-treated PBS (DEPCPBS) at room temperature (RT) for 5 min, and they were washed in DEPC-PBS followed by 0.1N HCl treatment at RT for 5 min. The sections were washed in DEPC-PBS again, and they were subjected to reverse transcription, probe ligation, and rolling circle amplification reactions. The RCA products were detected by hybridization of AF750-labeled detection oligo and decoded through four cycles of barcode sequencing, each involving a base-specific incorporation of fluorescence dyes (A = Cy5, C = Texas Red, G = Cy3, T = AF488), imaging, and removing of the incorporated dyes.

### Imaging setup and image analysis

Images were acquired using a Zeiss Axio Imager Z2 epifluorescence microscope (Zeiss Oberkochen, Germany), equipped with a × 40 objective. A series of images (10% overlap between two neighboring images) at different focal depths was obtained, and the stacks of images were merged into a single image thereafter using the maximum-intensity projection (MIP) in the Zeiss ZEN software. After exporting images in .tif format, images were aligned between cycles and then stitched together using the MIST algorithm. The stitched images were then re-tiled into multiple smaller images, henceforth referred to as tiles. Tiled DAPI images were segmented with standard watershed segmentation. The tiled images with RCA products were top-hat filtered, followed by initial nonlinear image registration, spot detection, fine image registration, crosstalk compensation, gene calling, and cell calling using the pciSeq pipeline (https://github.com/acycliq/full_coronal_section [8]).

### Genes and cell typing

The analysis pipeline further assigns genes to cells and then cells to cell types. It leverages the area, the global *x* and *y* coordinates, and the unique cellular identifier of the segmented cells as well as the global *x* and *y* coordinates of the genes. The assignment is done using a probabilistic framework based on single-cell RNA sequencing data (probabilistic cell typing by in situ sequencing (pciSeq)). Here, our 124 targeted genes for the ISS experiments were selected from the single-cell RNA sequencing data from [2] that was also used for cell typing. The gene panel selection algorithm and the cell typing algorithm have been described in [8] and showed that for hippocampal interneurons, around 70 genes were needed for fine classification. For the oligodendrocytes, we performed a similar analysis; however, more genes were needed for a fine classification in pciSeq to reach the same levels of accuracy. The ranking of the oligodendrocyte genes was based on the median gene expression values, i.e., the median expression values per gene per cell type from the single-cell RNA sequencing experiments from [2] were calculated. We also applied differential gene expression analysis to the single-cell RNA sequencing data using Scanpy [45]. Some genes were manually curated, analogous to [8], i.e., genes that have been used in classical immunohistochemistry experiments have been included. For example, *Plp1* was added as a PAN marker gene, and we only considered cells expressing *Plp1* for analysis (to minimize the risk of potential false classification). We opted to include 8–10 genes per cell type; however, since many OL-associated genes are shared between OL populations, we did not always find distinctive genes.

### Data analysis

The highest probability in the pie charts was used to define the final oligodendrocyte type (winner-takes-all principle), and only cells containing *Plp1* were kept for downstream analysis. Out of the 4820 OLs in P10 tissue, only 58 had a probability smaller than 50%, and only 5 OLs had a probability smaller than 40%. Out of the 8088 OLs in P20 tissue, only 117 had a probability smaller than 50%, and only 13 OLs had a probability smaller than 40%. Out of the 16,268 OLs in P60 tissue, only 109 had a probability smaller than 50%, and only 5 OLs had a probability smaller than 40%. The downstream analysis was carried out with custom-made scripts in MATLAB and Python (https://github.com/Moldia).

Comparisons of cell counts between conditions (CTX and CC or P20 and P60) were calculated by normalizing the cell counts for each sample first. We calculated both, the cell occurrences per *Plp1+* cells (%) and the cell occurrences per tissue area (cells/mm^2^). For comparisons of cell densities, we calculated the rate of change of the oligodendrocyte densities as *ratio = 100/(condition X for cell_type A/condition Y for cell_type A)*. For comparisons of relative cell occurrences, the cell counts for each oligodendrocyte population were divided by the total number of *Plp1*+ cells per sample in order to account for the differences in sample size and cell densities (values between 0 and 100). The ratios of the relative cell counts were calculated as *ratio = 100/(condition X for cell_type A/condition Y for cell_type A)* with *condition X* denoting the larger cell count and *condition Y* denoting the smaller cell count (*X, Y = {CTX, CC, P20, P60}*). The ratio was subtracted from 100 to express increases, i.e., if *condition X* is 30 and *condition Y* is 10, the heatmap shows a value of 66.66%, meaning that the increase from *condition Y* to *condition X* is 20 (or 66.66% of *condition X*). Bar plots show the mean ± SEM (standard error of mean). Density plots were generated using the MATLAB function ksdensity and registered to Allen Mouse Brain Atlas using a custom-made script similar to [46].

Nearest neighbor analysis was performed by first partitioning the cell maps into regions using the MATLAB function voronoi. The MATLAB function delaunayTriangulation (dual to the Voronoi diagrams) was used to create an object of neighbors of points, and the MATLAB function nearestNeighbor was used to capture the Euclidean distance of these points. Both the distances (in μm) and relative abundances (in %) of nearest neighbors were calculated and visualized as bar plots and heatmaps, respectively. Thereby, the absolute abundances of nearest neighbors were divided by the total number of nearest neighbors per cell type. To assess the arrangement of the OLs, we applied neighborhood enrichment analysis similar to [18], which utilizes a permutation-based test and compares the cell type labels with a random configuration of labels by maintaining the positional information.

Statistical comparisons were determined using two-tailed Student’s paired *t* test, and to account for multiple comparisons, the data were analyzed using ANOVA and post hoc test with Tukey correction (*p < 0.05, **p < 0.01, ***p < 0.001, and ****p < 0.0001). Data throughout the study is presented as mean ± SEM.

## Supplementary Information

**Additional file 1: Fig. S1.** Cell type probabilities and genes per cell for P10, P20 and P60. **a** Violin plots of the calling probabilities from pciSeq, assigning cell types by the highest probability values, respectively (top: P10; middle: P20; bottom: P60; CTX and CC joined) (*n*=4 P10 tissue sections, *n*=6 P20 tissue sections, *n*=6 P60 tissue sections). **b** Violin plots of the number of total transcripts detected in the oligodendrocyte lineage cells and VLMCs (top: P10; middle: P20; bottom: P60; CTX and CC joined). **c** The number of distinct genes in oligodendrocyte lineage cells and VLMCs (top: P10; middle: P20; bottom: P60; CTX and CC joined).

**Additional file 2: Fig. S2.** Gene expression per cell type. **a** Heatmaps showing the log 2 expression per gene. The individual genes assigned to the cell populations are ranked by their expression values with the leftmost gene always having the highest expression for the assigned cell population (top: P10; middle: P20; bottom: P60; CTX and CC joined). **b** The 10 most differentially-expressed genes for each cell population. The x-axis shows the ranking, the y-axis the score from Wilcoxon rank-sum.

**Additional file 3: Fig. S3.** Representative cell maps of brain. **a** Cell maps of OL populations for P10 CTX. The cells are assigned by the highest probability and colored accordingly. The scale bar is 500 μm. **b** Same as (**a)** for P20 CTX. **c** Same as (**a)** for P60 CTX.

**Additional file 4: Fig. S4.** Probabilities of cell types for P10, P20 and P60. Heatmaps of the probability distribution in the pie charts that are not the highest probability (mean confusion matrix; top: P10; middle: P20; bottom: P60; CTX and CC joined).

**Additional file 5: Fig. S5.** Oligodendrocyte density per area for P10, P20 and P60 CTX (GM) and CC (WM). **a** Number of oligodendrocytes per area (in cells/mm^2^) for P10 CTX (top) and P10 CC (bottom) (*n*=4 P10 tissue sections). **b** Same as (**a)** for P20 (*n*=6 P20 tissue sections). **c** Same as (**a)** for P60 (*n*=6 P60 tissue sections).

**Additional file 6: Fig. S6.** Representative cell maps of spinal cord. **a** Cell maps of OL populations for P60 GM. The cells are assigned by the highest probability and colored accordingly. The scale bar is 100 μm. **b** Same as (**a)** for 60 WM.

## Data Availability

All data generated or analyzed during this study are included in this published article, its supplementary information files, and publicly available repositories. Single-cell RNA sequencing cell type definitions are from [2] and can be accessed through http://linnarssonlab.org/oligodendrocytes. The pciSeq pipeline is available at https://github.com/acycliq/full_coronal_section, the custom-made scripts for the data analysis, and the in situ sequencing data (cell-by-gene matrices) at https://github.com/Moldia.
