# Supplementary figures and images for: Spatial and temporal heterogeneity in the lineage progression of fine oligodendrocyte subtypes
Source: BMC Biol. 2022 May 25;20:122. doi: 10.1186/s12915-022-01325-z (PMC9131697; doi:10.1186/s12915-022-01325-z)

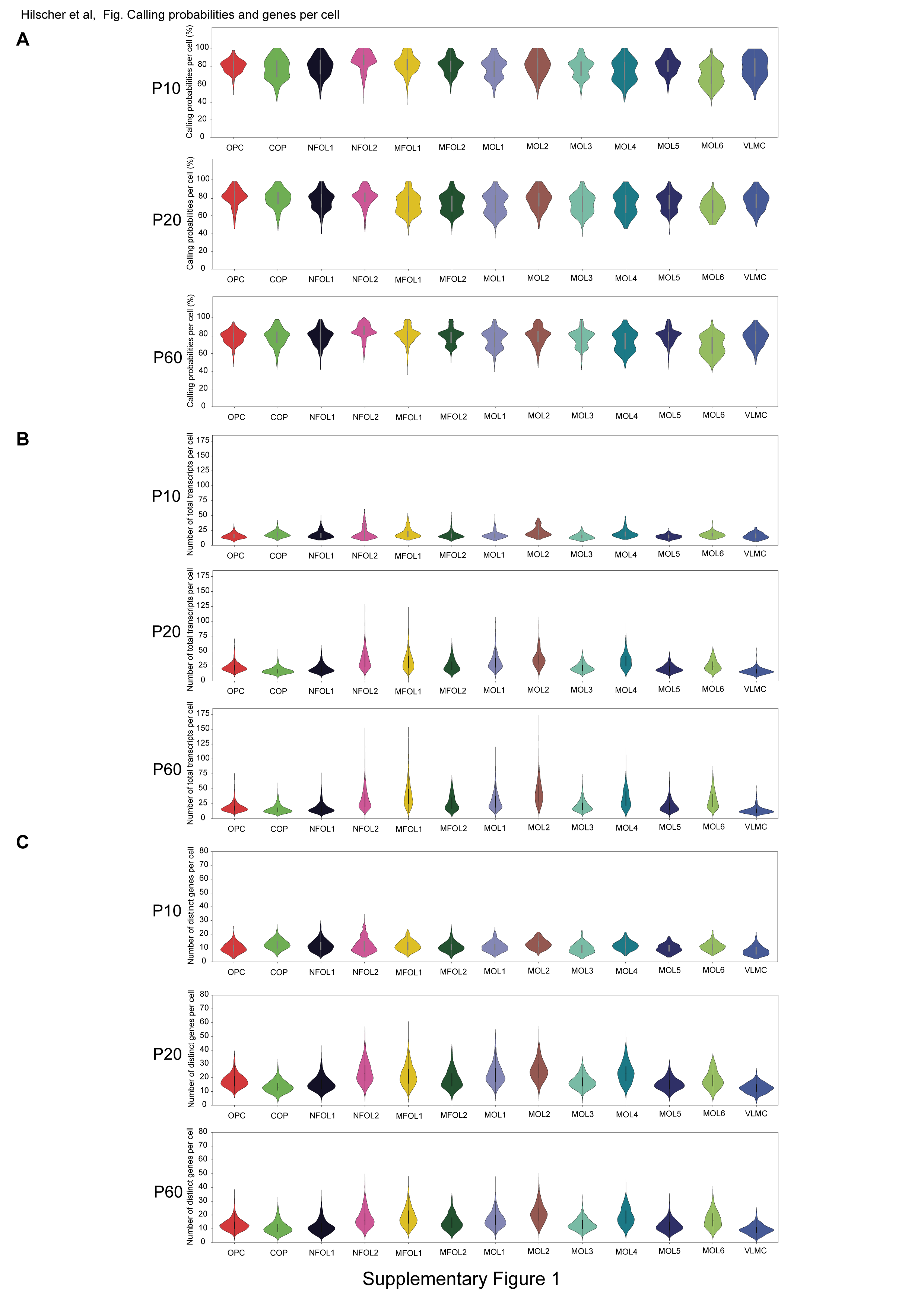

Supplement: Supplementary file 1 — Additional file 1: Fig. S1. Cell type probabilities and genes per cell for P10, P20 and P60. a Violin plots of the calling probabilities from pciSeq, assigning cell types by the highest probability values, respectively (top: P10; middle: P20; bottom: P60; CTX and CC joined) (n=4 P10 tissue sections, n=6 P20 tissue sections, n=6 P60 tissue sections). b Violin plots of the number of total transcripts detected in the oligodendrocyte lineage cells and VLMCs (top: P10; middle: P20; bottom: P60; CTX and CC joined). c The number of distinct genes in oligodendrocyte lineage cells and VLMCs (top: P10; middle: P20; bottom: P60; CTX and CC joined). [file 12915_2022_1325_MOESM1_ESM.tif]

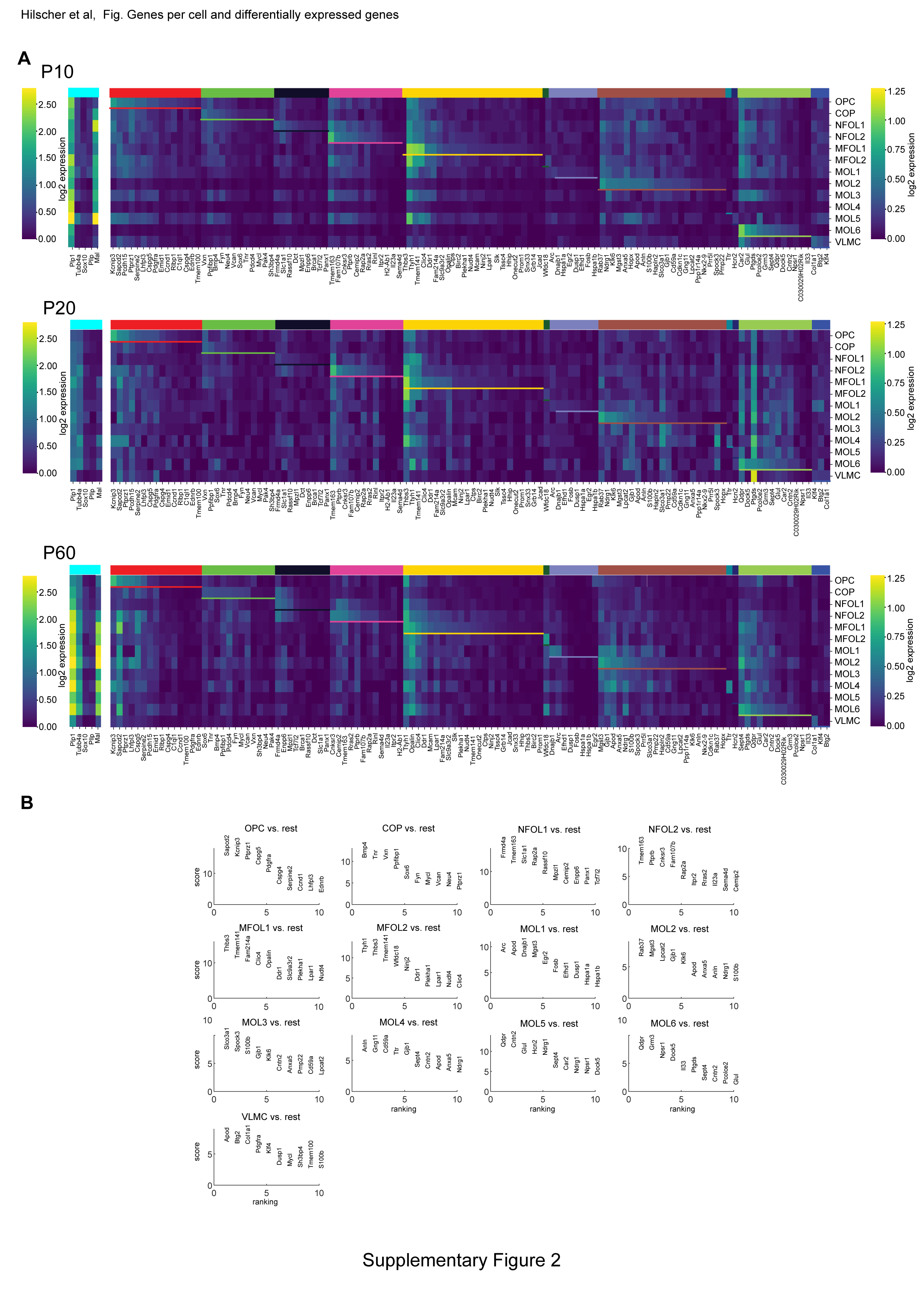

Supplement: Supplementary file 2 — Additional file 2: Fig. S2. Gene expression per cell type. a Heatmaps showing the log 2 expression per gene. The individual genes assigned to the cell populations are ranked by their expression values with the leftmost gene always having the highest expression for the assigned cell population (top: P10; middle: P20; bottom: P60; CTX and CC joined). b The 10 most differentially-expressed genes for each cell population. The x-axis shows the ranking, the y-axis the score from Wilcoxon rank-sum. [file 12915_2022_1325_MOESM2_ESM.tif]

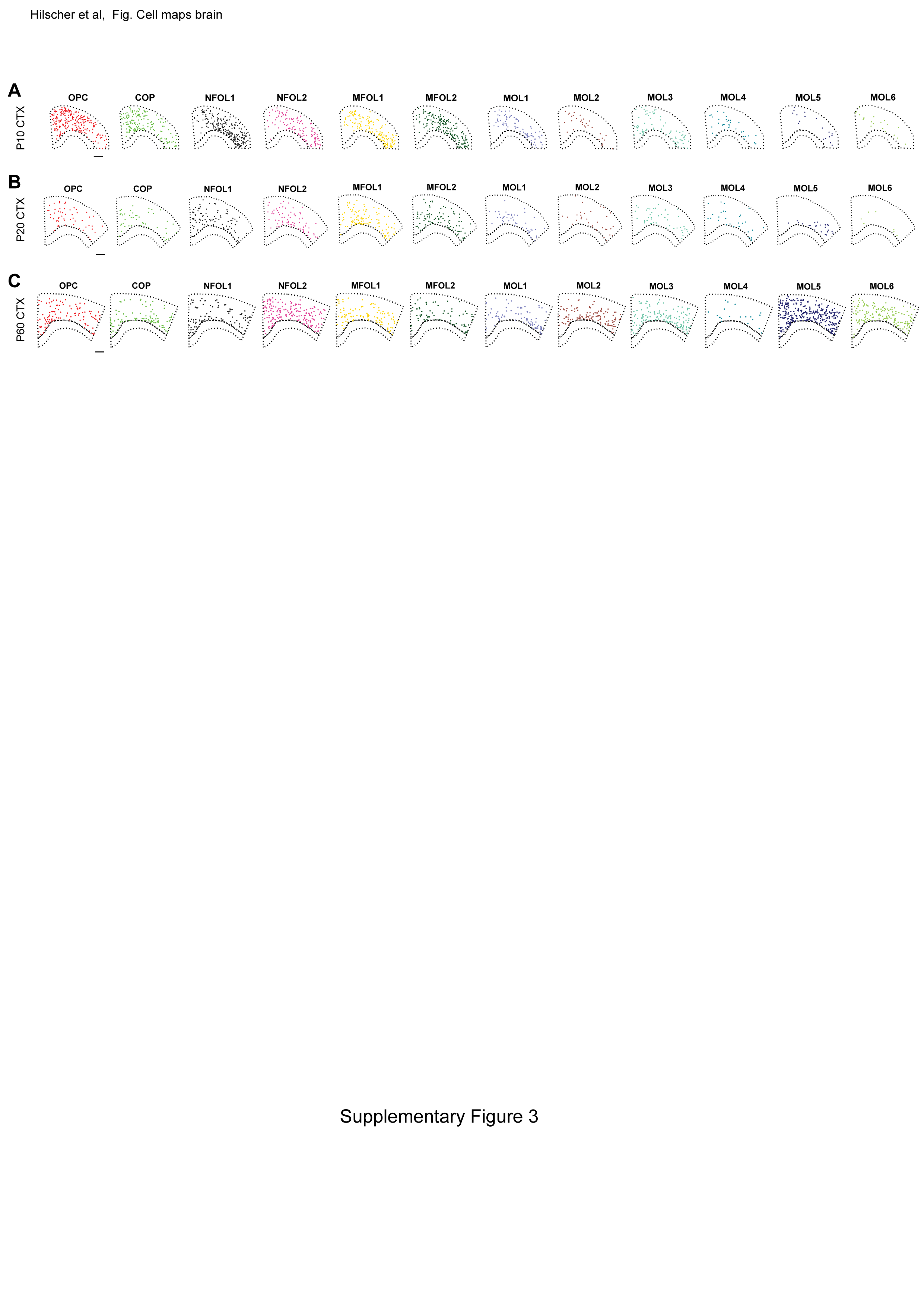

Supplement: Supplementary file 3 — Additional file 3: Fig. S3. Representative cell maps of brain. a Cell maps of OL populations for P10 CTX. The cells are assigned by the highest probability and colored accordingly. The scale bar is 500 μm. b Same as (a) for P20 CTX. c Same as (a) for P60 CTX. [file 12915_2022_1325_MOESM3_ESM.tif]

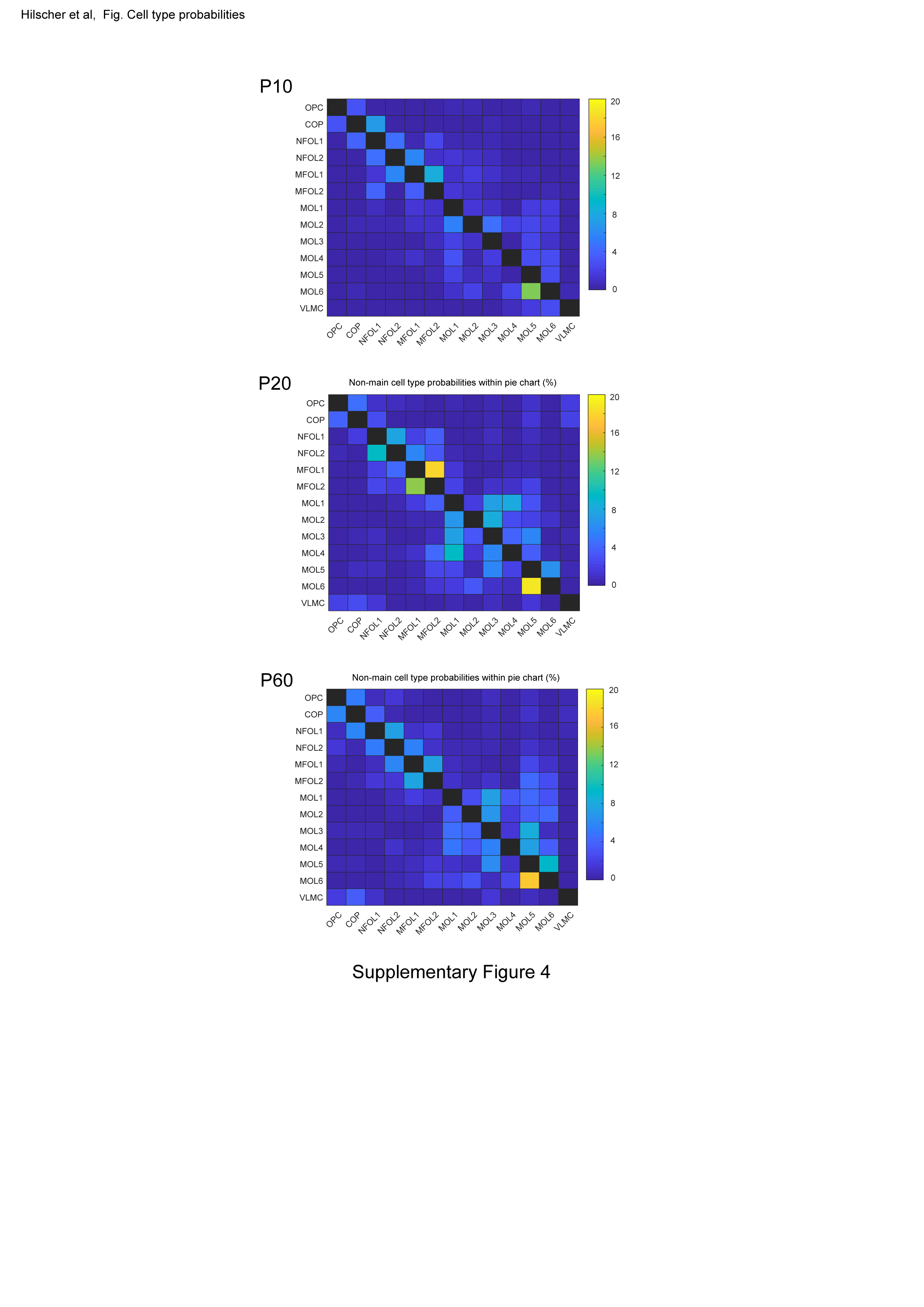

Supplement: Supplementary file 4 — Additional file 4: Fig. S4. Probabilities of cell types for P10, P20 and P60. Heatmaps of the probability distribution in the pie charts that are not the highest probability (mean confusion matrix; top: P10; middle: P20; bottom: P60; CTX and CC joined). [file 12915_2022_1325_MOESM4_ESM.tif]

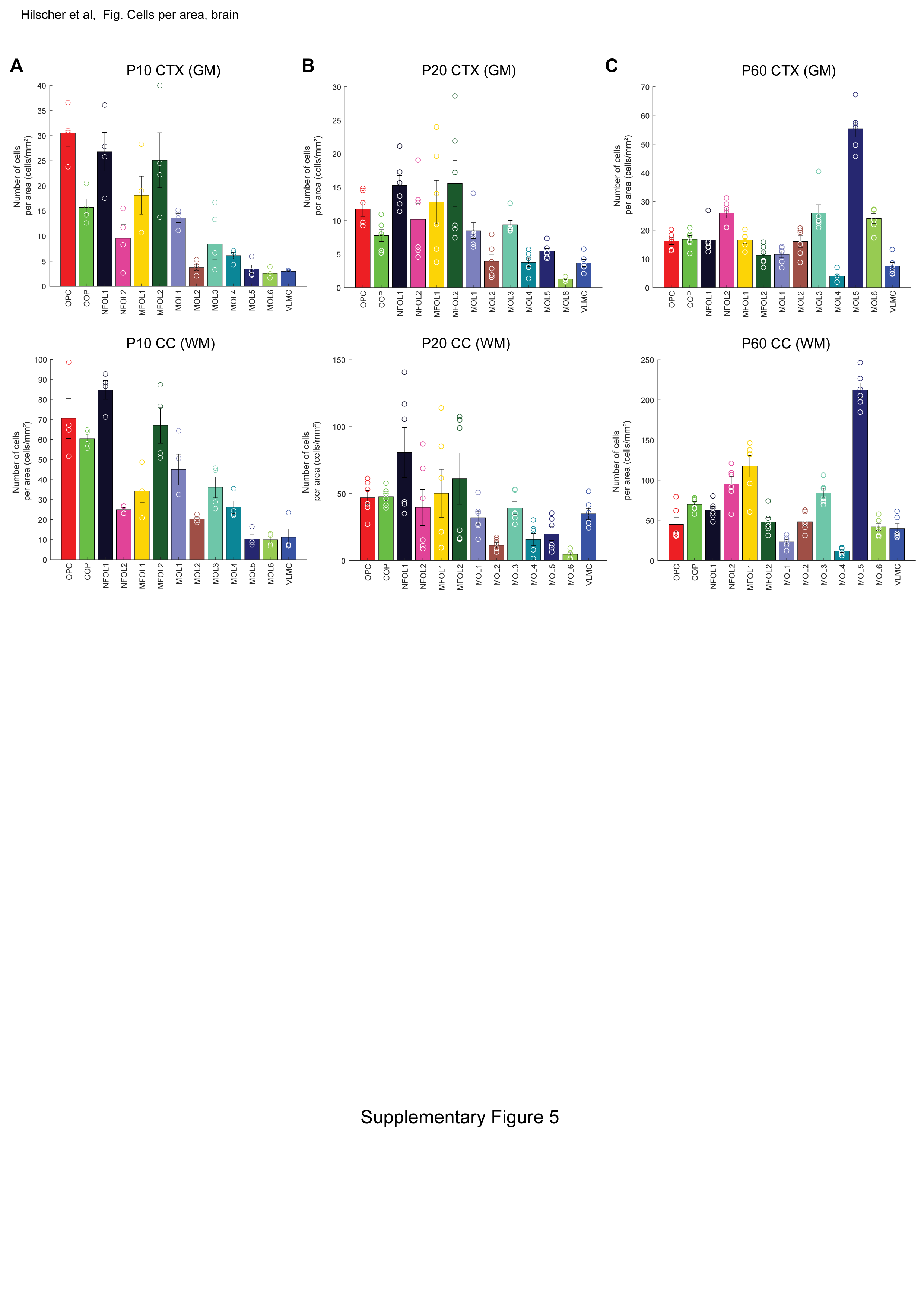

Supplement: Supplementary file 5 — Additional file 5: Fig. S5. Oligodendrocyte density per area for P10, P20 and P60 CTX (GM) and CC (WM). a Number of oligodendrocytes per area (in cells/mm2) for P10 CTX (top) and P10 CC (bottom) (n=4 P10 tissue sections). b Same as (a) for P20 (n=6 P20 tissue sections). c Same as (a) for P60 (n=6 P60 tissue sections). [file 12915_2022_1325_MOESM5_ESM.tif]

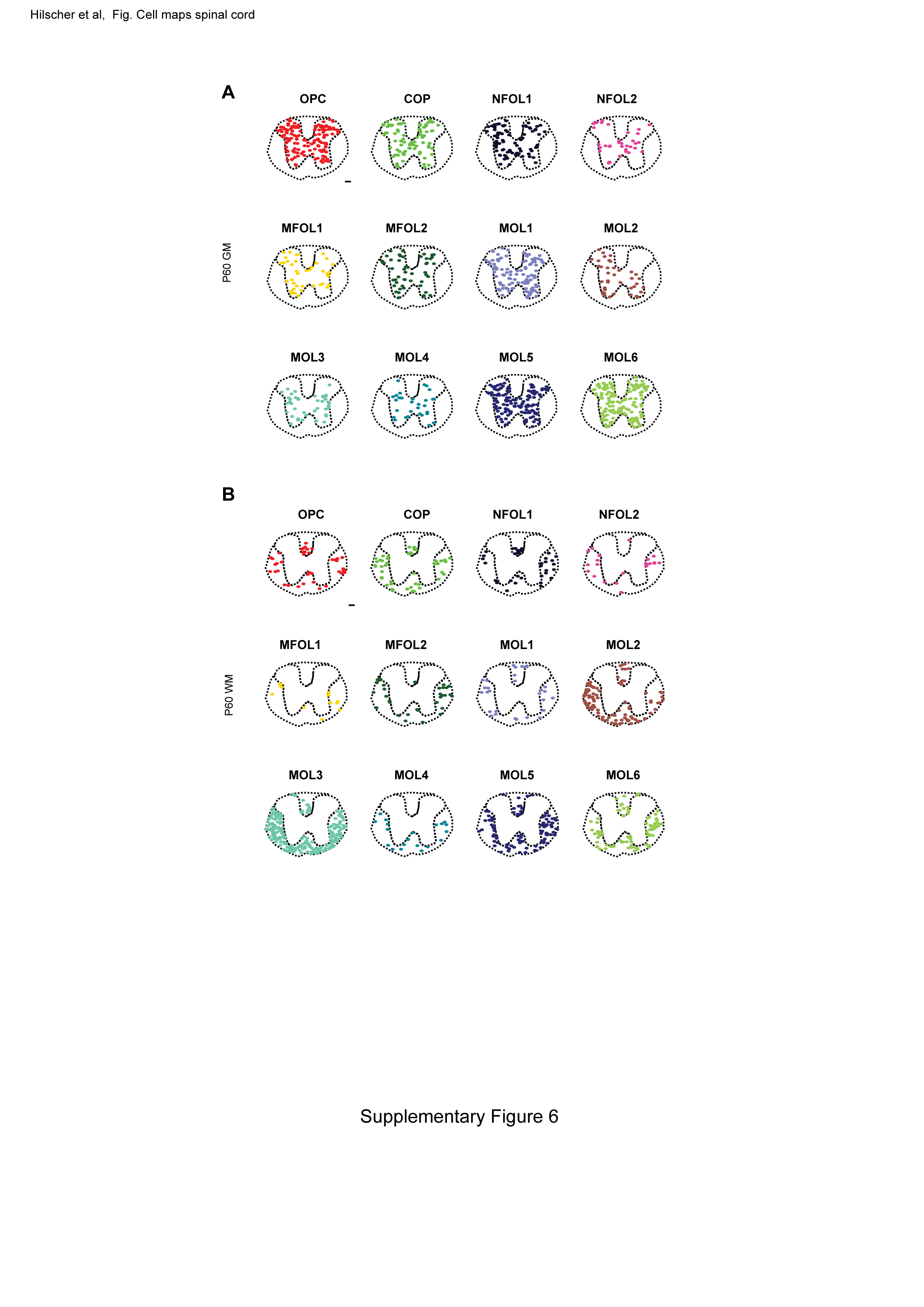

Supplement: Supplementary file 6 — Additional file 6: Fig. S6. Representative cell maps of spinal cord. a Cell maps of OL populations for P60 GM. The cells are assigned by the highest probability and colored accordingly. The scale bar is 100 μm. b Same as (a) for 60 WM. [file 12915_2022_1325_MOESM6_ESM.tif]
